# Hydroxamic Acid-Based Histone Deacetylase (HDAC) Inhibitors Bearing a Pyrazole Scaffold and a Cinnamoyl Linker

**DOI:** 10.3390/ijms20040945

**Published:** 2019-02-21

**Authors:** Chiara Zagni, Andrea Citarella, Mahjoub Oussama, Antonio Rescifina, Alessandro Maugeri, Michele Navarra, Angela Scala, Anna Piperno, Nicola Micale

**Affiliations:** 1 Department of Drug Sciences, University of Catania, Viale A. Doria 6, 95125 Catania, Italy; chiarazagni@gmail.com (C.Z.); arescifina@unict.it (A.R.); 2 Department of Chemical, Biological, Pharmaceutical, and Environmental Sciences, University of Messina, Viale F. Stagno D’Alcontres 31, 98166 Messina, Italy; acitarella@unime.it (A.C.); amaugeri@unime.it (A.M.); mnavarra@unime.it (M.N.); ascala@unime.it (A.S.); apiperno@unime.it (A.P.); 3 Higher Institute of Applied Sciences and Technology of Mahdia, University of Monastir, Sidi Massa-oud, Hiboun Mahdia Tunisian 5100, Tunisia; oussamamahjoub1993@gmail.com; 4 Consorzio Interuniversitario Nazionale di ricerca in Metodologie e Processi Innovativi di Sintesi (C.I.N.M.P.S.), Via E. Orabona, 4, 70125 Bari, Italy

**Keywords:** HDAC inhibitors, hydroxamic acid, *N*^1^-aryl-pyrazole, *N*^1^-H-pyrazole, antiproliferative activity

## Abstract

Genetic abnormalities have been conventionally considered as hallmarks of cancer. However, recent studies have demonstrated that epigenetic mechanisms are also implicated in the insurgence and development of cancer. Patterns of the epigenetic component include DNA methylation and histone modifications. Acetylation of histones is controlled by histone acetyltransferases (HATs) and histone deacetylases (HDACs). Imbalance of these two enzymatic systems is known to be a key factor in tumor progression. Because HDACs have been found to function incorrectly in cancer, various HDAC inhibitors (HDACIs) are being investigated to act as cancer chemotherapeutics. Herein, we report the synthesis, docking studies and biological activity of a series of hydroxamic acid-based HDACIs bearing an *N*^1^-aryl or *N*^1^-H pyrazole nucleus as surface recognition motif and a cinnamoyl group as a linker to the hydroxamic acid zinc-binding group (ZBG). Some of the tested compounds exhibited inhibitory properties towards HDACs and antiproliferative activity against neuroblastoma SH-SY5Y tumor cell line both at micromolar concentrations.

## 1. Introduction

The incidence of malignancies has dramatically risen in recent years. According to the recent report (2018) of the World Health Organization, cancer is currently the second leading cause of death worldwide. Therefore, the identification and improvement of new therapeutic strategies are urgently needed [1]. In this regard, it is well known the role of various histone deacetylase (HDAC) isoforms which act as epigenetic modulators of gene transcription and whose inhibition is crucial to identify novel anticancer agents. The epigenetic process is in large part coordinated through the control of the chromatin structure, and it is highly regulated by post-translational modifications of histones, around which nuclear DNA is wrapped, and the DNA itself. In particular, it has been shown that hyperacetylation of histones leads to transcriptional activation of suppressed genes, whereas hypoacetylation is correlated with reduced gene expression [2]. Imbalance of the activity of both histone acetyltransferase (HAT) and HDAC is strictly related to the insurgency and progression of tumors. Therefore, these epigenetic markers have been the focus of drug discovery during the last decade. In particular, histone deacetylase inhibitors (HDACIs) represent a promising new class of compounds for the treatment of cancer [3,4,5].

**Figure 1:**
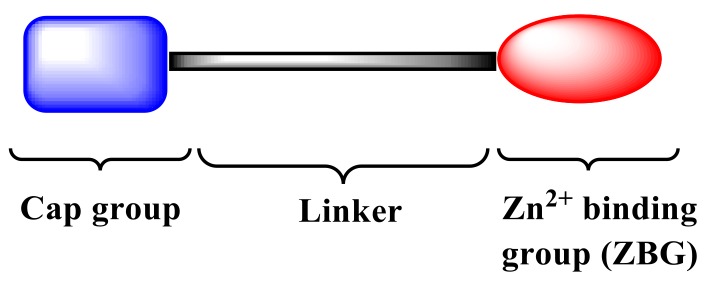
Pharmacophoric model of the designed histone deacetylases inhibitors (HDACIs).

However, there are no selective HDACIs approved for clinical use in oncology, albeit some of them reached Phase I or early Phase II trials [6]. The US FDA has approved only a few pan-HDACIs, and most of them are hydroxamic acid-based compounds which retrace the classic pharmacophoric model of these type of enzyme inhibitors consisting in: (i) a terminal metal-binding moiety (i.e., hydroxamic acid; ZBG) that coordinates the cation Zn^2+^ within the HDAC active site; (ii) a hydrophobic capping group that interacts with the external domain of the enzyme and regulates the interaction between the HDAC and substrate; (iii) a linker (generally a chain of six methylene groups) that sets the metal-binding moiety and the capping group for interactions within the binding site [7]. (Figure 1).

On the basis of these fruitful features, we report herein the synthesis of two sets of new potential HDACIs in which the hydroxamic acid moiety was maintained as a steady point for the zinc-binding. The cinnamoyl group was used to resemble the classical six methylene groups linker, and a pyrazole scaffold (joined to the linker throught an amide connection unit) was elected as capping group to achieve potency and possibly selectivity for the different HDAC isoforms. The cinnamoyl hydroxamic acid is a well-known and fruitfully exploited chemical fragment for HDACs inhibition [8,9,10,11,12,13]. Indeed, some cinnamoyl hydroxamates such as Belinostat (peripheral T-cell lymphoma) and Panobinostat (multiple myeloma) have been already approved by the FDA for the anticancer combination therapy. On the other hand, the pyrazole scaffold has been recently reassessed as a valid surface recognition motif [14,15].

## 2. Results and Discussion

### 2.1. Chemistry

**Figure 2:**
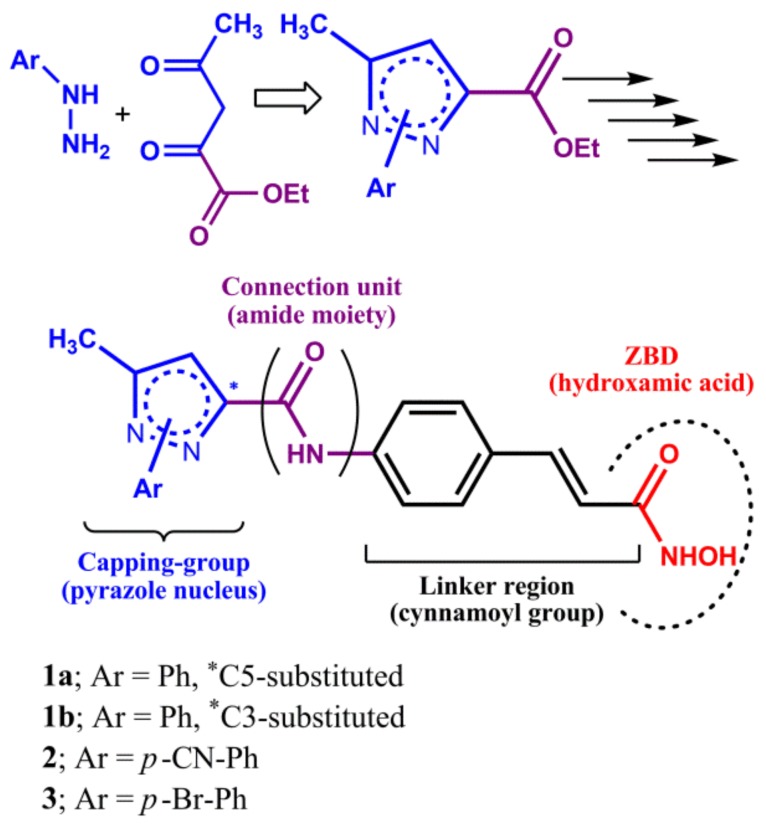
Design of the first set of HDACIs (*N*^1^-aryl-pyrazole derivatives). For synthetic details, see Scheme S1.

The first synthetic strategy (Figure 2 and Scheme S1) was intended to obtain an *N*^1^-aryl-pyrazole capping group and entails the condensation of different aryl-hydrazines with ethyl 2,4-dioxovalerate [16]. The resulting *N*^1^-aryl-pyrazole scaffolds bearing an ester moiety at C3 or C5 (two isomers were isolated because the condensation is not regiospecific. See Supporting Information for details) were conjugated after hydrolysis with the cinnamoyl linker using an amide moiety under standard coupling conditions. Finally, the terminal ester moiety of each adduct capping group/linker was converted into the corresponding hydroxamic acid moiety by a three-step procedure (Figure 2 and Scheme S1) [17].

**Figure 3:**
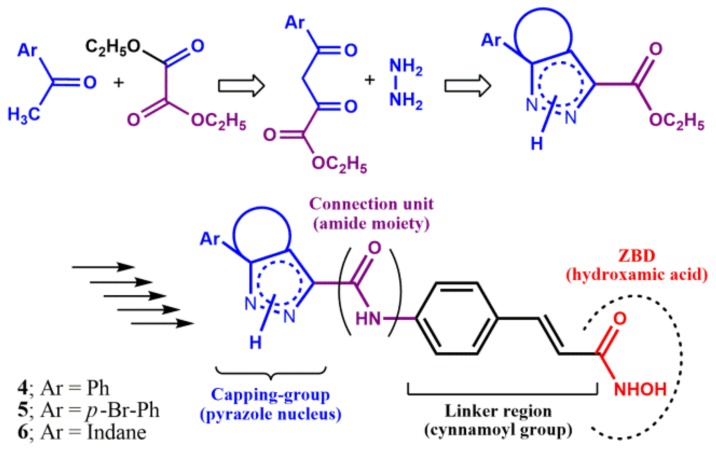
Design of the second set of HDACIs (aryl-substituted *N*^1^-H-pyrazole derivatives). For synthetic details, see Schemes S2 and S3.

The second synthetic strategy (Figure 3 and Schemes S2 and S3) was designed to obtain the *N*^1^-H pyrazole derivatives substituted at (C4)C5 with an aryl group, and entails the formation of α,γ-diketoesters by Claisen condensation of various aryl methyl ketones with diethyl malonate followed by cyclization with hydrazine [18]. The desired new pyrazole capping groups were then transformed into the final compounds according to the previous synthetic methodology. The structures of new final compounds, as well as of each intermediate, were determined by analytical and spectroscopic data (see Supplementary Materials).

### 2.2. Biological Evaluation

The *N*^1^-phenylpyrazole derivatives **1a** (the isomer with the connection unit at C5) and **1b** (the isomer with the connection unit at C3) were evaluated for their ability to inhibit the HDAC activity to have a preliminary insight about the SAR of this set of compounds. The commercially available assay kit HDAC Inhibitor Drug Screening Kit (Biovision) was employed for this test and Trichostatin A (TSA), which is a natural pan-HDACI, was used as positive control. As can be seen from the data reported in Table 1, only the derivative **1a** turned out to be active (IC_50_ = 7.1 µM). Therefore, the development of the other two derivatives of this class, i.e., the *p*-CN-phenyl derivative **2** and the *p*-Br-phenyl derivative **3**, was focused on the single isomer C5-substituted. As for **1a**, the detected inhibitory activity of **2** and **3** was in the low micromolar range (6.3 µM and 1.6 µM, respectively; Table 1). Regarding the set of *N*^1^-H pyrazole derivatives, only the indane derivative 6 displayed fair HDAC inhibitory activity (IC_50_ = 46.5 µM), whereas the *p*-Br-phenyl derivative 5 turned out to be inactive (IC_50_ > 500 µM) and the phenyl-derivative 4 showed only a modest inhibition on the intended target (IC_50_ = 155.2 µM).

**Figure 4:**
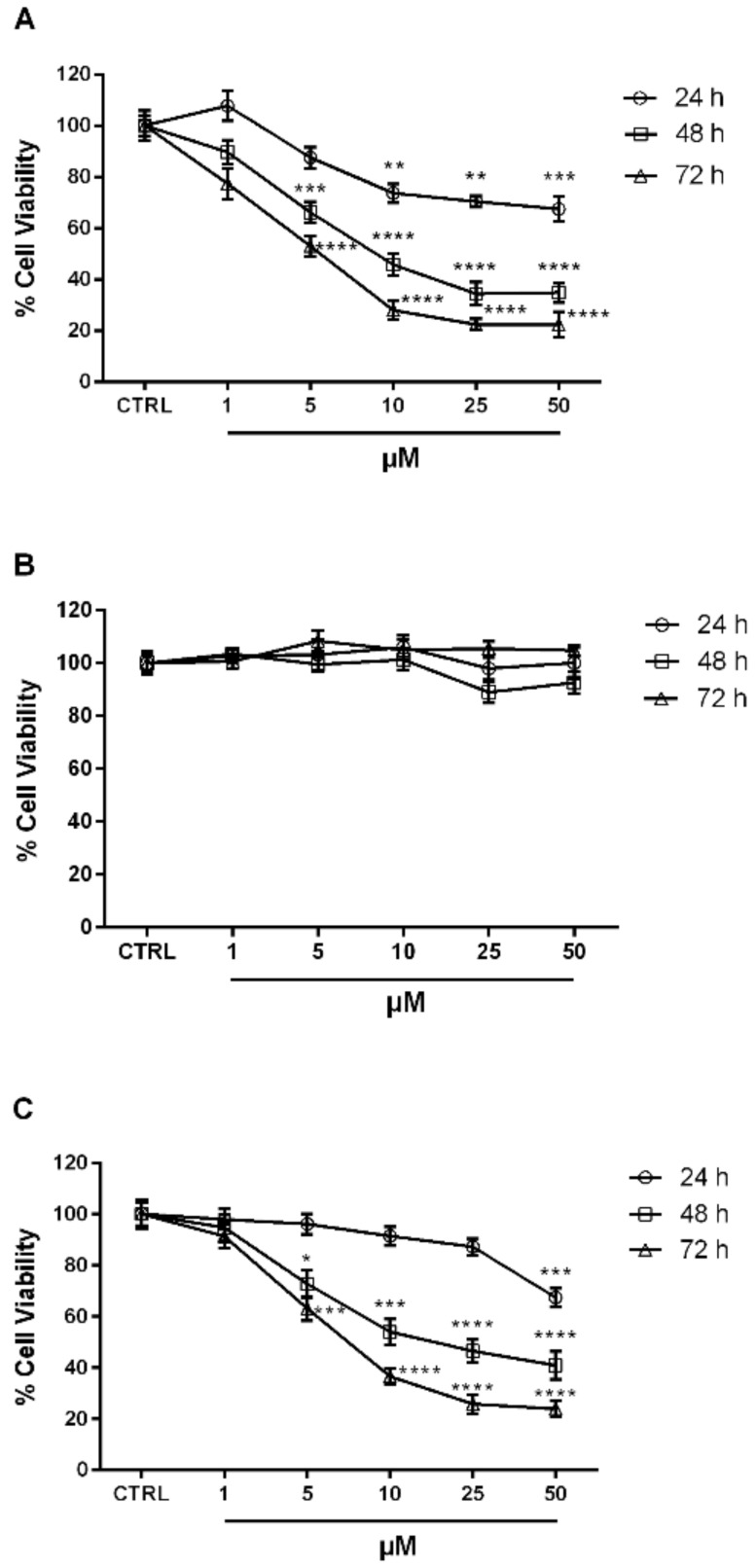
Compounds **1a** (**A**) and **3** (**C**) exerted antiproliferative effect on SH-SY5Y cells exposed to increasing concentration of synthesized compounds for 24, 48, and 72 h. No antiproliferative effect was observed for compound **2a** (**B**). Each value is the mean ± S.E.M. of three experiments performed eight times. **** *p* < 0.0001, *** *p* < 0.001, ** *p* < 0.01, * *p* < 0.05 vs. CTRL.

Finally, the compounds which turned out to be the most active in the enzyme assay, i.e., the *N*^1^-aryl-pyrazole derivatives **1a**, **2** and **3**, were also tested in vitro by the MTT [3-(4,5-dimethylthiazole-2-yl)-2,5-diphenyltetrazolium bromide] test to evaluate their antiproliferative activity against the neuroblastoma SH-SY5Y tumor cell line [20,21]. The treatment of the SH-SY5Y cells with growing concentrations (1–50 µM) of **1a** (Figure 4A) and **3** (Figure 4C) significantly reduced cell proliferation, both reaching more than 75% of inhibition at 25 µM after 72 h (**** *p* < 0.0001 vs. CTRL). Compounds **1a** and **3** showed an IC_50_ of 5.34 and 5.61 µM, respectively. None of the tested concentrations of **2a**, instead, turned out to be effective (Figure 4B).

### 2.3. Molecular Docking

**Figure 5:**
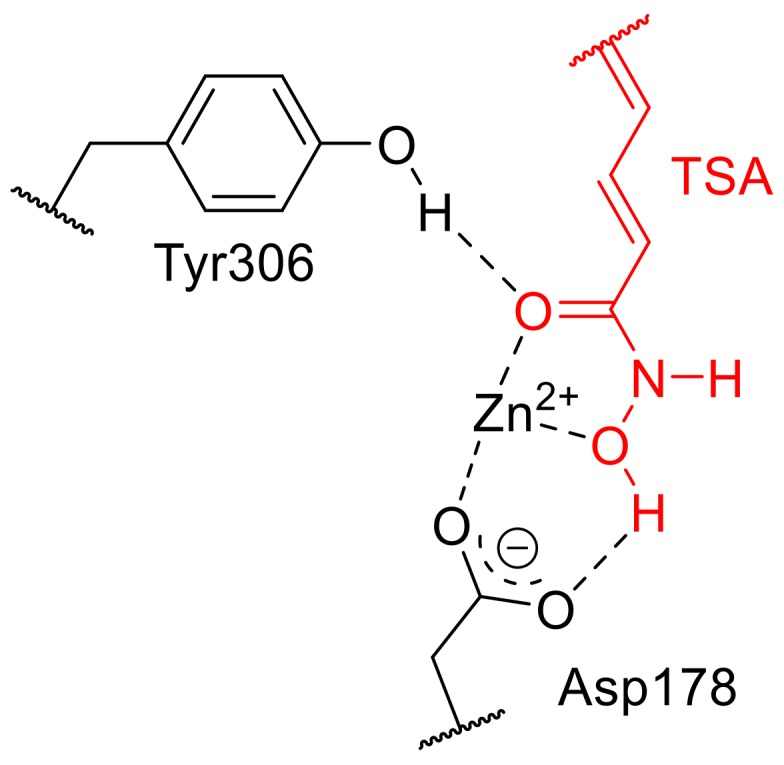
Interactions of TSA in the co-crystallized structure with the human HDAC8 (PDB ID: 1T64) and chelation with Zn^2+^.

In order to explain the different ability of the compounds **1**–**6** to inhibit HDAC enzymes, we performed a docking simulation using the active site of the three-dimensional structure of the HDAC8 enzyme. In fact, except for HDAC8, functional HDACIs were found as high-molecular-weight multiprotein complexes, and most purified recombinant HDACIs are enzymatically inactive [22]. Therefore, HDAC8 is the best model among mammalian HDACIs if we look at it from a structural biology perspective. Thus, looking at the pose of TSA in the co-crystallized structure with the human HDAC8 (PDB ID: 1T64) and considering that hydroxamic acid group chelates the zinc ion in a bidentate fashion and forms hydrogen bonds with Tyr306 and Asp178 (Figure 5) we docked compounds **1**–**6** starting from this pose.

**Figure 6:**
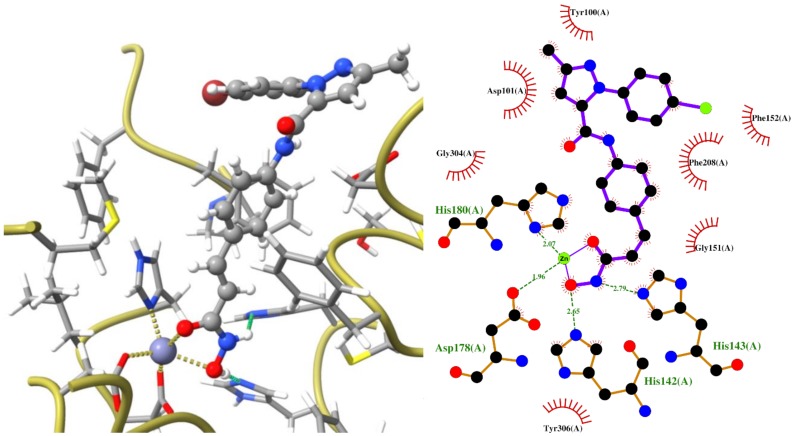
3D and 2D view of the interactions for 3 inside the binding pocket of HDAC8 (PDB ID: 1T64); lengths in Å. The 3D plot was generated by UCSF ChimeraX (release 0.8, Resource for Biocomputing, Visualization, and Informatics, San Francisco, CA, USA) [26]; hydrogen bonds are shown as green dotted lines, while coordination bonds are in yellow. The 2D plot was generated by LigPlot^+^ (2.1, EMBL-EBI, Wellcome Genome Campus, Hinxton, Cambridgeshire, United Kingdom) [27]; hydrogen bonds are shown as green dotted lines, while the spoked arcs represent residues making nonbonded contacts with the ligand.

The molecular docking simulations of all new compounds showed that the trend of activity is maintained along the series but with about a 10-fold underestimated value [23,24,25]. However, the same order is maintained for the TSA and suberoylanilide hydroxamic acid (SAHA), the two most famous HDACIs, chosen as reference compounds (Table 1) and used to validate the docking procedure. The 3D and 2D sketch of the docked pose for the best-scored compound **3** (Figure 6) highlight that this compound chelates the Zn^2+^ bidentately, as expected, whereas a hydrogen bond is established between hydroxamic NH hydrogen and His143, while another with OH hydrogen and His142. Moreover, a series of *π*–*π* stacked interactions occur between the aromatic rings and Phe152, Phe208, His180, and Tyr100; furthermore, a *π*-anion interaction involves the pyrazole ring and Asp101 (Figure 6).

## 3. Materials and Methods

### 3.1. Chemistry

#### 3.1.1. General Experimental Information

All reagents used for the development of the HDACIs, including *N*-(3-dimethylaminopropyl)-*N*′-ethyl carbodiimide hydrochloride (EDCI), 1-hydroxybenzotriazole hydrate (HOBt) and various hydrazines, as well as all synthesis solvents and deuterated solvents employed for NMR analysis, were purchased from Sigma-Aldrich/Fluka (Milano, Italia) and used without further purification.

NMR spectra were recorded by means of a Varian Gemini 500 MHz (^1^H) e 125 MHz (^13^C) instrument or, alternatively, a Brüker Avance III 400 MHz (^1^H), using CDCl_3_, Acetone-*d*_6_, CD_3_OD, D_2_O e DMSO-*d*_6_ as solvents depending on the solubility of the compounds; the chemical shifts (*δ*) were provided in ppm using TMS as internal standard and coupling constants (*J*) in Hertz (Hz). The patterns of splitting were described as singlet (s), doublet (d), double doublet (dd), triplet (t), quartet (q), multiplet (m), and broad singlet (bs). NMR data of each intermediate of synthesis are reported only within the Supporting Information.

Melting points of the various synthetic intermediates and final compounds were determined using a “BUCHI Melting Point B-545 Apparatus” and are incorrect. Elemental analysis of the final compounds was performed using a “Carlo Erba Model 1106” instrument (Elemental Analyzer for C, H, and N) and the obtained results were in the range of ±0.4% compared with the theoretical values.

Thin layer chromatography (TLC) was performed on Merck 60 F_254_ plates (Merck KGaA, Darmstadt, Germany). For the column chromatography Macherey-Nagel 60 M (0.040–0.063 mm) silica gel was used.

#### 3.1.2. General Procedure for the Synthesis of the *N*^1^-phenyl-pyrazole Scaffold

In a round bottom flask, 400 mg of ethyl 2,4-dioxovalerate (0.35 mL, 2.53 mmol) were dissolved in ethanol (~40 mL) and reacted under reflux for ~3h with 1 equivalent of phenyl-hydrazine (0.25 mL, 2.53 mmol) in the presence of a catalytic amount of HCl_conc._.

Then, the solvent was removed under reduced pressure, and the resulting crude product was dissolved with brine and extracted thrice with EtOAc. The collected organic phases were dried over anhydrous Na_2_SO_4_, filtered and evaporated under vacuum conditions to afford 655 mg of crude oil. The latter was purified by column chromatography using a mixture of petroleum ether/ethyl acetate 8:2 as eluent to afford isomers **11a** and **11b** in a 1.2:1 molar ratio due to the fact that the cyclization of the starting α,γ-diketoester with phenylhydrazine is not regiospecific.

From the mere ^1^H NMR analysis, it turned out to be difficult to ascribe with certainty the stereochemistry of the two isomers; therefore, further experiments of bidimensional NMR spectroscopy (2D NOESY) were performed before proceeding separately with the design and synthesis of the *N*^1^-aryl-pyrazole HDACIs.

From these experiments (see Supporting Information) emerged that the only difference between the two isomers is the detection of a long-range coupling between the –CH_3_ group at 2.31 ppm and the aromatic hydrogens in the range 7.45–7.39 ppm of the phenyl ring observed for the isomer **11b**, which clearly indicates the proximity of these groups and the subsequent ascription therein reported.

#### 3.1.3. General Procedure for the Hydrolysis of the *N*^1^-phenyl-pyrazole Scaffold

To free the carboxy group of the pyrazole derivative in such a way to allow its subsequent coupling with the selected cinnamoyl linker, 245 mg (1.06 mmol) of 11a were dissolved in EtOH (~30 mL) and placed in an ice bath under vigorous stirring. Then, a solution of LiOH 1N was added dropwise to the reaction mixture, which was left to react at room temperature and continuously monitored by TLC (EP/EtOAc 8:2). After ~5 h, the chromatographic check indicated the completion of the reaction. Thus, the solvent was removed under reduced pressure, and the residue was solubilized in H_2_O and extracted with a small amount of ethyl ether to eliminate the residual organic impurities. The aqueous phase containing the desired compound (as Li^+^ salt) was acidified with HCl and re-extracted several times with ethyl acetate. The combined organic phases were dried over anhydrous Na_2_SO_4_, filtered, and evaporated to afford the product as yellow-brownish solid which was purified by trituration with a mixture of ethyl ether/petroleum ether. With the same procedure was obtained the isomer **14b**.

#### 3.1.4. General Procedure for the Coupling between the *N*^1^-phenyl-pyrazole Scaffold (Cap) and the Cinnamoyl Linker

The acid pyrazole derivative **14a** (202 mg, 1.0 mmol) was placed in a flask and solubilized in CH_2_Cl_2_ (~20 mL). The reaction mixture was cooled with an ice bath and left under vigorous stirring for ~20 min. Then, HOBt (203 mg, 1.5 mmol), EDCI (288 mg, 1.5 mmol) and the linker (ethyl 4-amino-cinnamate, 191 mg, 1.0 mmol) were added in sequence, and the reaction mixture was left at room temperature overnight. Work-up: The synthesis mixture was diluted with CH_2_Cl_2_ and washed in sequence with a solution of citric acid (10%), NaHCO_3_ (5%) and brine. The organic phase was dried over anhydrous Na_2_SO_4_, filtered and removed in vacuo. The resulting crude was purified by column chromatography (eluting solution EP/EtOAc 7:3) to afford the desired product **17a**. The coupling of the isomer **14b** was performed with the same procedure, starting from 167 mg (0.83 mmol).

#### 3.1.5. General Procedure for the Hydrolysis of the Adduct *N*^1^-phenyl-pyrazole (Cap)/Cinnamoyl Linker

251 mg (0.67 mmol) of **17a** were dissolved in THF (~30 mL) and the resulting solution was cooled with an ice bath before adding LiOH 1N dropwise. The progress of the reaction was monitored by TLC (disappearance of the starting material) and turned out to be completed in ~6h. Afterward, the reaction solution was concentrated with the rotary evaporator and the residue was diluted with H_2_O, acidified with HCl and extracted several times with ethyl acetate. The combined organic phases were dried over anhydrous Na_2_SO_4_, filtered and removed under vacuum to afford a crude product which was purified by column chromatography using petroleum ether/ethyl acetate 1:1 + 2% of formic acid as eluent to obtain the desired product. In the same way, was performed the hydrolysis of the CAP-linker **17b** starting from 195 mg (0.52 mmol).

#### 3.1.6. General Procedure for the Synthesis of the Final Cinnamoyl Hydroxamic Acid with CAP *N*^1^-phenyl-pyrazole (**1a**,**1b**)

In order to obtain the final hydroxamic zinc-binding domain (ZBD), the terminal group –COOH of the adduct CAP-linker **20a** (202 mg, 0.58 mmol) was joined with 1 equivalent of an O-protected derivative of the hydroxylamine [O-(*tert*-butyldimethylsilyl)hydroxylamine] (86 mg, 0.58 mmol) easily to be de-protected in mild conditions, using EDCI (167 mg, 0.87 mmol) as a coupling reagent in CH_2_Cl_2_. The formation of the desired new adduct was detected by the TLC [R*_f_* = 0.35 (TLC: Hexane/EtOAc 1:1)] and quickly checked by NMR (data not shown). After the removal of the solvent, the resulting crude product was employed as such in the next synthetic step. Thus, the crude product was reacted with a solution of TFA (25% in CH_2_Cl_2_) at 0 °C for ~5h to free the hydroxamic acid moiety and eventually the solvent was removed under vacuum. After these two consecutive synthetic steps, the pure final product (**1a**) was obtained by trituration of the resulting crude product with ethyl ether.

**(*E*)-*N*-(4-(3-(hydroxyamino)-3-oxoprop-1-enyl)phenyl)-3-methyl-1-phenyl-1H-pyrazole-5-carboxamide (1a);**^1^H NMR (500 MHz, CD_3_OD): *δ* ppm 7.63–7.40 (m, 10H, 9ArH + Ar–CH=), 6.79 (s, 1H, H-4), 6.40 (d, 1H, *J* = 15.9 Hz, =CHCONHOH), 2.36 (s, 3H, Pyr–CH_3_).

^13^C NMR (125 MHz, CD_3_OD): *δ* ppm 165.0 (–*C*ONHOH), 158.9 (–CONH), 149.1 (C5), 139.9 (ArCH=), 139.4, 137.9, 131.1, 128.6, 128.0, 127.8, 126.1, 124.5, 120.2, 116.2 (C4), 108.6 (=CHCO), 13.2 (Pyr–CH_3_). R*_f_* = 0.51 (TLC: EtOAc/MeOH 8:2); yellowish powder. M.p. = 103–107 °C. Yield: 101 mg (48%). Anal. Calcd for C_20_H_18_N_4_O_3_: C, 66.29; H, 5.01; N, 15.46. Found: C, 66.35; H, 5.03; N, 15.50.

HRMS-EI (*m*/*z*) [M]^+^ calcd for C_20_H_18_N_4_O_3_ 362.1379, found 362.1381.

With the same procedure was obtained the final product **1b** starting from 123 mg (0.35 mmol) of the related acid adduct 20b, 52 mg (0.35 mmol) of TBDMSiO-NH_2_ and 102 mg (0.53 mmol) of EDCI.

**(*E*)-*N*-(4-(3-(hydroxyamino)-3-oxoprop-1-enyl)phenyl)-5-methyl-1-phenyl-1H-pyrazole-3-carboxamide (1b);**^1^H NMR (500 MHz, CD_3_OD): *δ* ppm 7.81 (d, 2H, *J* = 8.4 Hz, ArH), 7.72 (d, 2H, *J* = 8.4 Hz, ArH), 7.65–7.54 (m, 5H, ArH), 7.53 (d, 1H, *J* = 13.8 Hz, Ar–CH=), 6.80 (s, 1H, H-4), 6.30 (d, 1H, *J* = 13.8 Hz, =CHCONHOH), 2.36 (s, 3H, Pyr–CH_3_).

^13^C NMR (125 MHz, CD_3_OD): *δ* ppm 165.2 (–CONHOH), 159.8 (–CONH), 148.1 (C3), 143.4 (ArCH=), 140.7, 139.6, 129.9, 129.3, 129.1, 128.4, 126.8, 125.4, 118.8, 117.1 (C4), 107.5 (=CHCO), 11.9 (Pyr-CH_3_). R*_f_* = 0.18 (TLC: EtOAc/MeOH 8:2); yellowish powder. M.p. = 103–107 °C. Yield: 45 mg (42%). Anal. Calcd for C_20_H_18_N_4_O_3_: C, 66.29; H, 5.01; N, 15.46. Found: C, 66.26; H, 4.99; N, 15.49.

HRMS-EI (*m*/*z*) [M]^+^ calcd for C_20_H_18_N_4_O_3_ 362.1379, found 362.1382.

#### 3.1.7. Synthesis of the Other Hydroxamic Acids with CAP *N*^1^-aryl-pyrazole (**2**,**3**)

Regarding the other *N*^1^-aryl-pyrazole derivatives (i.e., *p*-cyanophenyl **2** and *p*-bromophenyl **3**), the design and synthesis were carried out only on the related pyrazole scaffolds of the isomer **12a** and **13a** in accordance with the preliminary biological data obtained with the *N*^1^-phenyl analogs **1a**,**b**. Analogous synthetic procedures were used, and the experimental data of each intermediate as well as of the final compounds are reported hereinafter.

**(*E*)-1-(4-cyanophenyl)-*N*-(4-(3-(hydroxyamino)-3-oxoprop-1-enyl)phenyl)-3-methyl-1H-pyrazole-5-carboxamide (2);**^1^H NMR (500 MHz, CD_3_OD): *δ* ppm 8.00–7.38 (m, 9H, 8ArH + Ar–CH=), 6.90 (s, 1H, H-4), 6.83 (d, 1H, *J* = 15.9 Hz, =CHCONHOH), 2.35 (s, 3H, Pyr–CH_3_).

^13^C NMR (125 MHz, CD_3_OD): *δ* ppm 164.9 (–CONHOH), 158.7 (–CONH), 150.3 (C5), 143.3 (Ar*C*H=), 132.7, 129.2, 128.9, 128.1, 126.4, 124.9, 124.1 120.3 (–*C*N), 117.8 (C4), 115.1 (*C*-CN), 110.6 (=CHCO), 11.2 (Pyr–CH_3_). Yellowish powder. R*_f_* = 0.12 (TLC: EtOAc/MeOH 8:2); M.p. = 110–114 °C. Anal. Calcd for C_21_H_17_N_5_O_3_: C, 65.11; H, 4.42; N, 18.08. Found: C, 65.18; H, 4.45; N, 18.13.

HRMS-EI (*m*/*z*) [M]^+^ calcd for C_21_H_17_N_5_O_3_ 387.1331, found 387.1333.

**(*E*)-1-(4-bromophenyl)-*N*-(4-(3-(hydroxyamino)-3-oxoprop-1-enyl)phenyl)-3-methyl-1*H*-pyrazole-5-carboxamide (3);**^1^H NMR (300 MHz, CD_3_OD): *δ* ppm 7.83–7.22 (m, 9H, 8Ar*H* + Ar–CH=), 6.83 (s, 1H, H-4), 6.40 (d, 1H, *J* = 15.9 Hz, =CHCONHOH), 2.35 (s, 3H, Pyr–CH_3_).

^13^C NMR (75 MHz, CDCl_3_): *δ* ppm 166.1 (–CONHOH), 158.7 (–CONH), 148.9 (C5), 143.4 (ArCH=), 140.7, 139.7, 137.8, 131.6, 130.5, 128.7, 126.1, 122.0 (C-Br), 119.8, 117.0 (C4), 109.2 (–CHCO), 13.0 (Pyr–*C*H_3_). R*_f_* = 0.13 (TLC: EtOAc/MeOH 8:2); M.p. = 103–107 °C. Pale pink solid. Anal. Calcd for C_20_H_17_BrN_4_O_3_: C, 54.44; H, 3.88; N, 12.70. Found: C, 54.52; H, 3.89; N, 12.73.

HRMS-EI (*m*/*z*) [M]^+^ calcd for C_20_H_17_BrN_4_O_3_ 440.0484, found 440.0486.

#### 3.1.8. Synthesis of the α,γ-Diketoester Intermediate **26** and **27**

The synthesis of the α,γ-diketoester intermediate **26** and **27** was carried out by Claisen condensation between acetophenones **23** and **24** and diethyl oxalate **25** in the presence of a strong base. Specifically, 750 mg of acetophenone **23** (6.24 mmol, 730 µL) were solubilized in ethanol (~50 mL) and then EtONa (850 mg, 12.5 mmol,) was added to the resulting solution. The temperature of reaction mixture was raised to ~50 °C for a few minutes, and afterward 1.83 g of diethyl oxalate (12.5 mmol, 1.6 mL) were added dropwise. The reaction was brought to reflux for ~3h and then dried under vacuum. The residue was solubilized in H_2_O acidified with HCl. The aqueous phase was extracted several times with ethyl acetate, and the combined organic phases were dried over anhydrous Na_2_SO_4_, filtered and concentrated under reduced pressure to afford a crude oil. The product (obtained in the enol form only) was purified by column chromatography using a mixture of petroleum ether/ethyl acetate 8:2 as mobile phase.

#### 3.1.9. Synthesis of *N*^1^H-pyrazole Scaffold C5-phenyl-substituted

The starting α,γ-diketoester **26** (1.17 g, 5.3 mmol) was dissolved in ethanol (~50 mL) and placed on a plate in an oil bath. Then, an excess (3 equivalent) of hydrazine hydrate (506 mg, 15.9 mmol, 499 µL) and a catalytic amount of HCl_conc._ were added to the solution and the reaction mixture was refluxed for ~3 h. Afterward, it was cooled, dried with a rotary evaporator, collected with brine, and extracted several times with ethyl ether. The combined organic phases were dried over anhydrous Na_2_SO_4_ and filtered. Then, the solvent was removed under reduced pressure, and the resulting crude oil was triturated with ethyl ether to provide a solid product which by NMR analysis turned out to be the hydrazide derivative of the initially designed compound. The filtered obtained by trituration with ethyl ether was purified by column chromatography using a mixture of n-hexane/ethyl acetate 7:3 as eluent to afford the expected product **28**. Beside of the expected ester **28**, the corresponding hydrazide derivative was recovered.

#### 3.1.10. Hydrolysis of the *N*^1^H-pyrazole Scaffolds C5-phenyl-substituted

As for the *N*^1^-aryl-pyrazole derivatives, the ester and the hydrazide moiety of the two *N*^1^H-pyrazole scaffolds must be hydrolyzed to –COOH group to carry out the coupling with the cinnamoyl linker.

**Route a**: 437 mg (2.0 mmol) of the ester derivative were dissolved in EtOH (~20 mL), placed in an ice bath under vigorous stirring and added dropwise with a solution of LiOH 1N. The reaction mixture was left to room temperature and monitored by TLC (n-Hexane/EtOAc 7:3). After ~5 h, the chromatographic check indicated the completion of the reaction (disappearance of the starting material). The reaction solvent was removed under vacuum, and the residue was collected with brine, acidified with HCl and extracted several times with ethyl acetate. The combined organic phases were dried over anhydrous Na_2_SO_4_ and filtered. Then, the solvent was removed under reduced pressure to afford the desired product as a whitish solid, which was purified by trituration with a mixture of ethyl ether/petroleum ether.

**Route b**: to a 538 mg (2.7 mmol) of the hydrazide derivative placed in a flask was added a mixture of HCl 6N/CH_3_COOH_gl._ (1:1; ~40 mL). The reaction was brought to reflux and left overnight under vigorous stirring. Work-up: The reaction solution was diluted with H_2_O and extracted several times with ethyl acetate. Then, the combined organic phases were dried over anhydrous Na_2_SO_4_ and filtered. The organic solvent was removed in vacuum and also in this case the desired product was obtained in a pure form by simple trituration of the residue with a mixture of ethyl ether/petroleum ether.

#### 3.1.11. Coupling Reaction between *N*^1^H-5-phenyl-pyrazole Scaffold (CAP) and Cinnamoyl Linker

The coupling reaction between the *N*^1^H-pyrazole CAP and the cinnamoyl linker and the pertaining workup were carried out with the same procedures used to obtain the *N*^1^-aryl-pyrazole derivatives 1–3, starting from 350 mg of acid 30 (1.86 mmol), 356 mg of linker (1.86 mmol), 377 mg of HOBt (2.79 mmol) and 535 mg of EDCI (2.79 mmol). Due to solubility issues, in some cases, a few drops of DMF were added to the reaction mixture. The desired pure adduct CAP-linker 32 was obtained by column chromatography using a mixture of CH_2_Cl_2_/EtOAc (8:2) as mobile phase.

#### 3.1.12. Hydrolysis of the Adduct *N*^1^H-pyrazole CAP/Cinnamoyl Linker

The hydrolysis of the ester moiety of the adduct *N*^1^H-pyrazole CAP/cinnamoyl linker 32 was performed in basic conditions using a solution of LiOH 1N in ethanol, starting from 383 mg (1.06 mmol) of product **34**. In addition, the work-up of the synthesis was performed with the same procedure.

#### 3.1.13. Synthesis of the Final Hydroxamic Acid with CAP *N*^1^H-5-phenyl-pyrazole (**4**)

The final conversion of the carboxylic acid moiety to hydroxamic acid moiety was carried out analogously to the *N*^1^-aryl-pyrazole derivatives **1**–**3** by means of a two steps synthetic procedure which entails the conjugation of a silyl derivative of the hydroxylamine (i.e., TBDMSiO–NH_2_) and the subsequent removal of the protecting silyl group by a solution of TFA (25%) in CH_2_Cl_2_, starting from 219 mg of acid 34 (0.66 mmol), 97 mg hydroxylamine derivative (0.66 mmol), 134 mg of HOBt (0.99 mmol) and 190 mg of EDCI (0.99 mmol).

**(*E*)-*N*-(4-(3-(hydroxyamino)-3-oxoprop-1-enyl)phenyl)-5-phenyl-1H-pyrazole-3-carboxamide (4);**^1^H NMR (500 MHz, DMSO-*d*_6_): *δ* ppm 13.89 (bs, 1H, PyrN*H*), 9.64 (bs, 1H, –CONH), 7.84 (d, 2H, *J* = 8.8 Hz, ArH), 7.75 (d, 2H, *J* = 7.9 Hz, ArH), 7.73 (d, 1H, *J* = 16.1 Hz, ArCH=), 7.64 (d, 2H, *J* = 8.8 Hz, ArH), 7.47 (t, 2H, *J* = 7.9 Hz, ArH), 7.38 (t, 1H, *J* = 7.9 Hz, ArH), 7.20 (s, 1H, PyrH-4), 6.62 (d, 1H, *J* = 16.1 Hz, =CHCO).

^13^C NMR (125 MHz, DMSO-*d*_6_): *δ* ppm 165.5 (–CONHOH), 161.0 (–CONH), 158.9 (ArCH=), 147.2 (C3), 141.1 (C5), 137.9, 131.3, 130.0, 129.5, 129.0, 125.9, 120.9, (=CHCO), 103.7 (C4). R*_f_* = 0.14 (TLC: 2% HCOOH in EtOAc/MeOH 8:2). Yellowish powder; M.p. = 254–258 °C. Yield: 87 mg (38%). Anal. Calcd for C_19_H_16_N_4_O_3_: C, 65.51; H, 4.63; N, 16.08. Found: C, 65.59; H, 4.60; N, 16.12.

HRMS-EI (*m*/*z*) [M]^+^ calcd for C_19_H_15_N_4_O_3_ 347.1144, found 347.1146.

#### 3.1.14. Synthesis of the Other Hydroxamic Acids with CAP *N*^1^H-aryl-substituted-pyrazole (**5**, **6**)

Regarding this second group of derivatives, the other compounds, which were designed and synthesized with the same procedure employed for the phenyl-derivative 4, are the *p*-bromophenyl (**5**) and the 1-indanone derivative (**6**), whose experimental data of the various intermediates as well as of the final compounds are hereinafter reported.

**(*E*)-5-(4-bromophenyl)-*N*-(4-(3-(hydroxyamino)-3-oxoprop-1-enyl)phenyl)-1H-pyrazole-3-carboxamide (5);**^1^H NMR (500 MHz, DMSO-*d*_6_): *δ* ppm 10.56 (bs, 1H, –CONH), 7.89 (d, 2H, *J* = 8.8 Hz, ArH), 7.80 (d, 2H, *J* = 8.3 Hz, ArH), 7.77 (d, 2H, *J* = 8.8 Hz, ArH), 7.64 (d, 2H, *J* = 8.3 Hz, ArH), 7.50 (d, 1H, *J* = 15.9 Hz, ArCH=), 7.19 (s, 1H, PyrH-4), 6.43 (d, 1H, *J* = 15.9 Hz, =CHCO).

^13^C NMR (125 MHz, DMSO-*d*_6_): *δ* ppm 168.4 (–CONHOH), 164.1 (–CONH), 148.0 (C3), 143.6 (ArCH=), 141.0 (C5), 132.1, 129.8, 129.4, 128.0, 125.1, 121.7 (C–Br), 120.5, 118.1, 107.1 (=CHCO), 104.2 (C4). R*_f_* = 0.17 (TLC: 2% HCOOH in EtOAc/CH_3_OH 8:2). Beige powder; M.p. = 222–224 °C. %). Anal. Calcd for C_19_H_15_BrN_4_O_3_: C, 53.41; H, 3.54; N, 13.11. Found: C, 53.47; H, 3.51; N, 13.15.

HRMS-EI (*m*/*z*) [M]^+^ calcd for C_19_H_14_BrN_4_O_3_ 425.0249, found 425.0248.

**(*E*)-*N*-(4-(3-(hydroxyamino)-3-oxoprop-1-enyl)phenyl)-1,4-dihydroindeno[1,2-*c*]pyrazole-3-carboxamide (6);**^1^H NMR (500 MHz, DMSO-*d*_6_): *δ* ppm 13.88 (bs, 1H, PyrNH), 9.98 (bs, 1H, –CONH), 8.00–7.80 (m, 1H, ArH), 7.67 (d, 2H, *J* = 8.8 Hz, ArH), 7.65 - 7.53 (m, 1H, ArH), 7.50 (d, 1H, *J* = 16.1 Hz, ArCH=), 7.36 (t, 1H, *J* = 7.9 Hz, ArH), 7.32 (d, 2H, *J* = 8.8 Hz, ArH), 7.29 (t, 1H, *J* = 7.9 Hz, ArH), 6.40 (d, 1H, *J* = 16.1 Hz, =CHCO), 3.69 (s, 2H, Ind–CH_2_).

^13^C NMR (125 MHz, DMSO-*d*_6_): *δ* ppm 169.5 (–CONHOH), 165.1 (–CONH), 155.0, 149.1, 143.9 (ArCH=), 141.5, 138.5, 130.8, 129.9, 129.4, 127.2, 126.9, 120.6, 119.4, 117.8 (=CHCO), 29.6 (Ind–CH_2_). R*_f_* = 0.58 (TLC: 2% HCOOH in EtOAc/CH_3_OH 8:2). Rusty powder; M.p. > 250 °C. Anal. Calcd for C_20_H_16_N_4_O_3_: C, 66.66; H, 4.48; N, 15.55. Found: C, 66.70; H, 4.46; N, 15.58.

HRMS-EI (*m*/*z*) [M]^+^ calcd for C_21_H_19_N_4_O_3_ 375.1457, found 375.1456.

### 3.2. Biological Activity

#### 3.2.1. HDAC Inhibitor Drug Screening Kit

HDAC-inhibitory activity was evaluated using a fluorometric HDAC assay kit (K340-100, Biovision, (Milpitas, CA, USA). The compounds, assay buffer, and HDAC fluorometric substrate were added to HeLa nuclear extract in a 96-well plate and incubated at 37 °C for 60 min. The reaction was stopped by adding a lysine developer, and the mixture was incubated for another 30 min at 37 °C. TSA was used as a positive control. Plates were read with excitation at 360 nm and emission at 450 nm. The data analysis was performed using GraphPad Prism 6.0.

#### 3.2.2. Cell Cultures and Treatment

Human neuroblastoma cell line SH-SY5Y was originally obtained from ATCC (Rockville, MD, USA). Cells were cultured as previously reported [20]. Briefly, we employed RPMI 1640 medium with 10% heat-inactivated fetal bovine serum (FBS), 2 mM l-glutamine, 1 mM sodium pyruvate, 100 IU/mL penicillin and 100 μg/mL streptomycin at 37 °C in a humidified atmosphere with 5% CO_2_. All the reagents for cell culture were from Carlo Erba (Milan, Italy). For biological investigations, compounds **1a**, **2** and **3** were solubilized in dimethyl sulfoxide (DMSO) to obtain stock solutions at a final concentration of 50 mM that were stored at −20 °C. Just prior use, aliquots were diluted in medium to the final concentrations ranging from 1 to 50 μM. The highest DMSO concentration here employed (0.1%) did not show any appreciable effect on cell proliferation.

#### 3.2.3. Antiproliferative Activity

The abovementioned compounds were evaluated by the MTT assay to assess their anti-proliferative activity, as reported by Ferlazzo et al. [21] Cells were seeded at a density of 5 × 10^3^ cell/well in 96-well plates and incubated overnight at 37 °C. In treated cells, the medium was then replaced with a fresh one in which the compounds were diluted from 1 to 50 µM. Untreated cells together with ones exposed to DMSO 0.1% served as control and solvent control, respectively. After incubation for 24, 48, and 72 h, plates were firstly centrifuged, then the medium was removed, and a solution of 3-(4,5-dimethylthiazole-2-yl)-2,5-diphenyltetrazolium bromide (MTT; Sigma-Aldrich, Milan, Italy) at a concentration of 0.5 mg/mL in fresh medium (w/o phenol red) was added. Plates were incubated for further 4h. Afterward, MTT solution was removed, and the obtained formazan crystals were solubilized in 100 µL of a lysis buffer consisting of HCl/isopropanol 0.1 N and regularly shaken. Optical density was measured employing a microplate reader (iMark™, Bio-Rad Laboratories, Milan, Italy) at 595 nm. All experiments were carried out in eight replicates for three times. Results are expressed as the percentage of viable cells compared to untreated cells.

#### 3.2.4. Statistical Analysis

Data were expressed as mean ± SEM and statistically evaluated for differences using one-way analysis of variance (ANOVA), followed by Tukey–Kramer multiple comparisons test (GraphPad Prism Software for Science).

### 3.3. Docking Studies

#### Docking Protocol

Macromolecule and ligands were prepared with Vega ZZ (3.1.1, Drug Design Laboratory, Milano, Italy) [23] assigning Gasteiger charges to the protein and AM1BCC ones to the ligand. Fine docking was performed by applying the Lamarckian genetic algorithm (LGA) implemented in AutoDock 4.2.6, The Scripps Research Institute, San Diego, California Jupiter, FL, US) [24] in combination with a specialized potential describing the interactions of zinc-coordinating ligands, named AutoDock4Zn (The Scripps Research Institute, San Diego, California Jupiter, FL, US) [25]. The ligand-centered maps were generated by the program AutoGrid (4.2.6, The Scripps Research Institute, San Diego, California Jupiter, FL, US) with a spacing of 0.375 Å and dimensions that encompass all atoms extending 5 Å from the surface of the ligand. All of the parameters were inserted at their default settings. In the docking tab, the macromolecule and ligand are selected, and GA parameters are set as ga_runs = 100, ga_pop_size = 150, ga_num_evals = 25,000,000, ga_num_generations = 27,000, ga_elitism = 1, ga_mutation_rate = 0.02, ga_crossover_rate = 0.8, ga_crossover_mode = two points, ga_cauchy_alpha = 0.0, ga_cauchy_beta = 1.0, number of generations for picking worst individual = 10.

Because no water molecule is directly involved in complex stabilization, they were not considered in the docking process. All protein amino acid residues were kept rigid whereas all single bonds of ligands were treated as fully flexible.

## 4. Conclusions

To sum up, two sets of new hydroxamic acid-based derivatives endowed with a pyrazole capping group and a cinnamoyl linker were designed, synthesized, and evaluated as HDACIs. All compounds having the *N*^1^-aryl-pyrazole scaffold as the capping group connected through the C5 at the cinnamoyl linker (i.e., **1a**, **2**, and **3**) showed promising inhibitory activity (IC_50_ range 1.3–6.3 µM) in the enzyme assay, whereas similar compounds having the *N*^1^-H-pyrazole scaffold (i.e., **4**, **5**, and **6**) as the capping group turned out to be much less active (**4** and **6**) or even inactive (**5**). Docking studies performed on these compounds using a simulation model of TSA co-crystallized with human HDAC8 isoform confirmed the effective insertion of the synthesized molecules within the tubular hydrophobic pocket of the target and the chelation of the terminal hydroxamic acid moiety with the Zn^2+^ enzyme cofactor. Overall, these studies also confirmed the trend observed in the enzyme assay. Besides, the results obtained from the evaluation of the antiproliferative activity of the *N*^1^-aryl-pyrazole derivatives **1a**, **2**, and **3** on neuroblastoma SH-SY5Y cells was in agreement with the enzyme inhibitory activity, except for the *p*-CN-phenyl derivative **2**, which turned out to be inactive. The other two compounds, instead (i.e., the phenyl derivative 1a and the *p*-Br–phenyl derivative **3**), reduced the cell proliferation with a dose- and time-dependent fashion, showing comparable IC_50_ values in the micromolar range.

Given these combined preliminary results on this type of derivatives, compound **3** can be considered as the lead structure for further developments.

## Figures and Tables

**Table 1:** HDAC inhibitory activity of compounds **1**–**6**, Trichostatin A (TSA) and suberoylanilide hydroxamic acid (SAHA) and calculated Δ*G*_bind_ and the corresponding *K*_i_.

Compd.	Inhibition at 10 μM (%) ^a^	Inhibition at 500 μM (%) ^a^	IC_50_ ±SD ^b^ (μM)	Calcd Δ*G*_bind_^c^	Calcd *K*_i_ (μM)
**1a**	—	86	7.1 ± 0.5	−7.96	1.45
**1b**	6	30	>500	−6.04	37.19
**2**	51	88	6.3 ± 0.3	−8.08	1.19
**3**	74	88	1.6 ± 0.2	−8.79	0.36
**4**	13	71	155.2 ± 2.8	−6.40	20.25
**5**	—	15	>500	−5.53	87.99
**6**	29	87	46.5 ± 1.5	−7.27	4.69
TSA ^d^	—	—	3.4 ± 0.2	−11.13	0.007
SAHA ^d^	—	—	—	−10.03	0.044

^a^ Standard deviations (SD) for the percent inhibition (mean of 3 independent measurements) were always <10%. ^b^ SD values. ^c^ kcal/mol. ^d^ Experimental *K*_i_ = 45 and 480 nM for TSA and SAHA, respectively [19].

## Supplementary Materials

Schematic procedures of synthesis (Schemes S1–S3), ^1^H and ^13^C NMR spectra, and other analytic data can be found at https://www.mdpi.com/1422-0067/20/4/945/s1.

## Abbreviations

EDCI	*N*-(3-dimethylaminopropyl)-*N*′-ethylcarbodiimide hydrochloride
HATs	histone acetyltransferases
HDACIs	histone deacetylase inhibitors
HDACs	histone deacetylases
HOBt	hydroxybenzotriazole
MTT	3-(4,5-dimethylthiazol-2-yl)-2,5-diphenyltetrazolium bromide
PDB	protein data bank
SAHA	suberoylanilide hydroxamic acid
TBDMSiO-NH2	*O*-(tert-butyldimethylsilyl)hydroxylamine
TSA	Trichostatin A
ZBD	zinc-binding domain
