# Supplementary material for: Hydroxamic Acid-Based Histone Deacetylase (HDAC) Inhibitors Bearing a Pyrazole Scaffold and a Cinnamoyl Linker
Source: Int J Mol Sci. 2019 Feb 21;20(4):945. doi: 10.3390/ijms20040945 (PMC6412695; doi:10.3390/ijms20040945)
Supplement: Supplementary file 1 [file ijms-20-00945-s001.pdf]

International Journal of
Molecular Sciences

MDPI

Supplementary Materials

Chemistry

Syntheses

General procedure for the synthesis of N¹-aryl-pyrazole derivatives

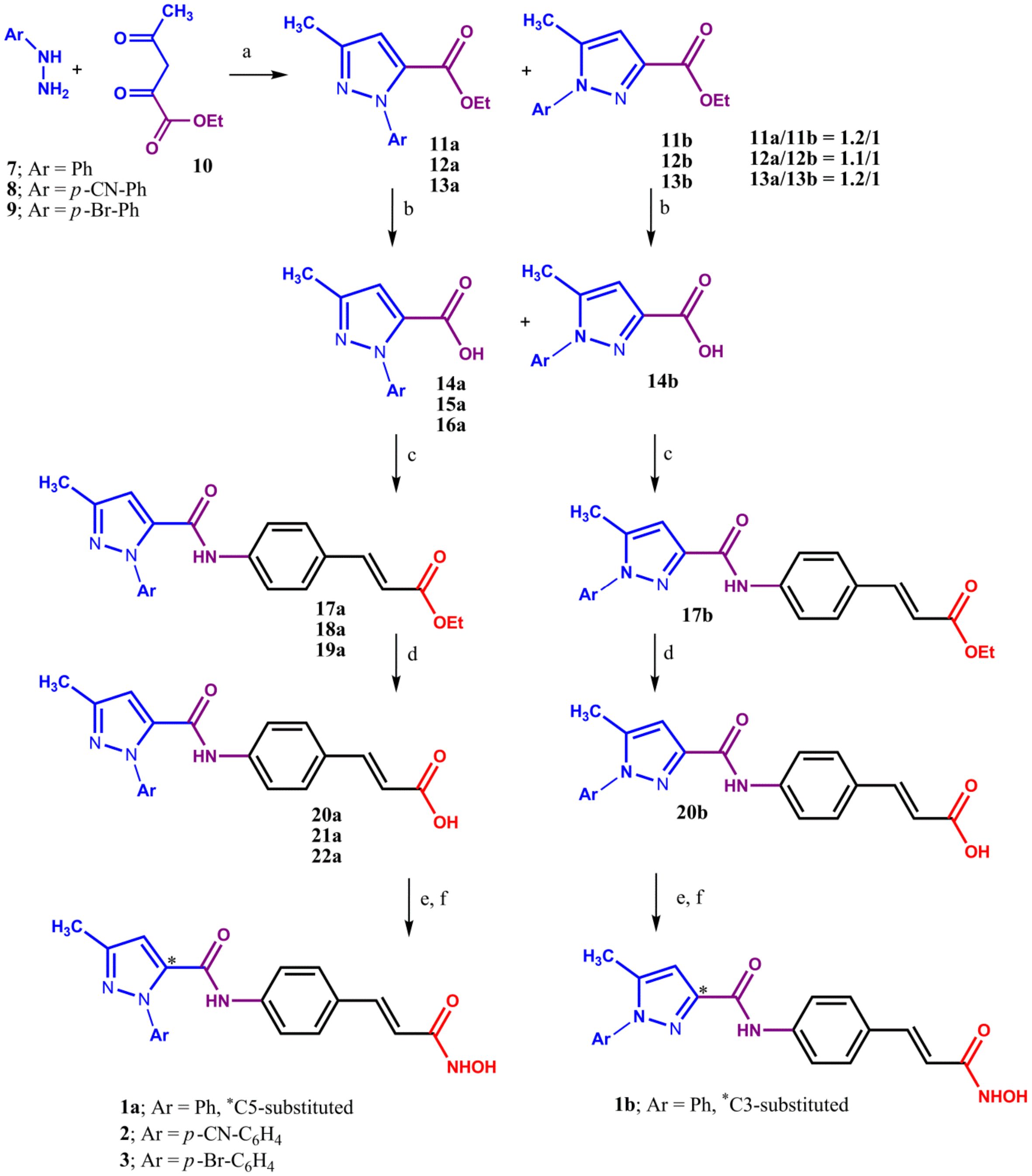

Scheme S1. Reagents and conditions: a) EtOH, $H^{+}_{cat.}$, Δ, 3h; b) LiOH 1N, EtOH, 0 °C to r.t., 5h; c) 4-NH_2PhCO_2Et, EDCI, HOBt, CH_2Cl_2, 0 °C to r.t., overnight; d) LiOH 1N, THF, 0 °C to r.t., 6h; e) TBDMSiO-NH_2, EDCI, CH_2Cl_2, 0 °C to r.t., overnight; f) TFA, CH_2Cl_2, 0 °C, 5h.

Formation of the N¹-phenyl-pyrazole scaffold

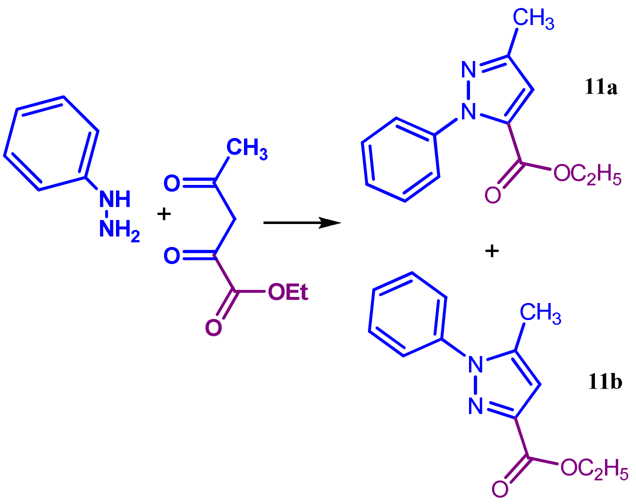

Reagents and conditions: EtOH, $H^{+}_{cat.}$, Δ, 3h.

11a: Ethyl 3-methyl-1-phenyl-1*H*-pyrazole-5-carboxylate

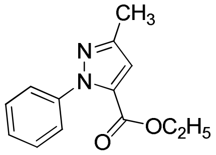

Mol. Wt.: 230.26

^{1}H NMR (500 MHz, $CDCl_3$) : *δ* ppm 7.42–7.38 (m, 5H, Ar), 6.80 (s, 1H, H-4), 4.20 (q, 2H, *J* = 7.1 Hz, -C*H*$_2$CH$_3$), 2.35 (s, 3H, Pyr-CH$_3$), 1.21 (t, 3H, *J* = 7.1 Hz, -CH$_2$C*H*$_3$). R_f = 0.74 (TLC: EP/EtOAc 8:2). Reddish-brown oil. Yield: 245 mg (42%).

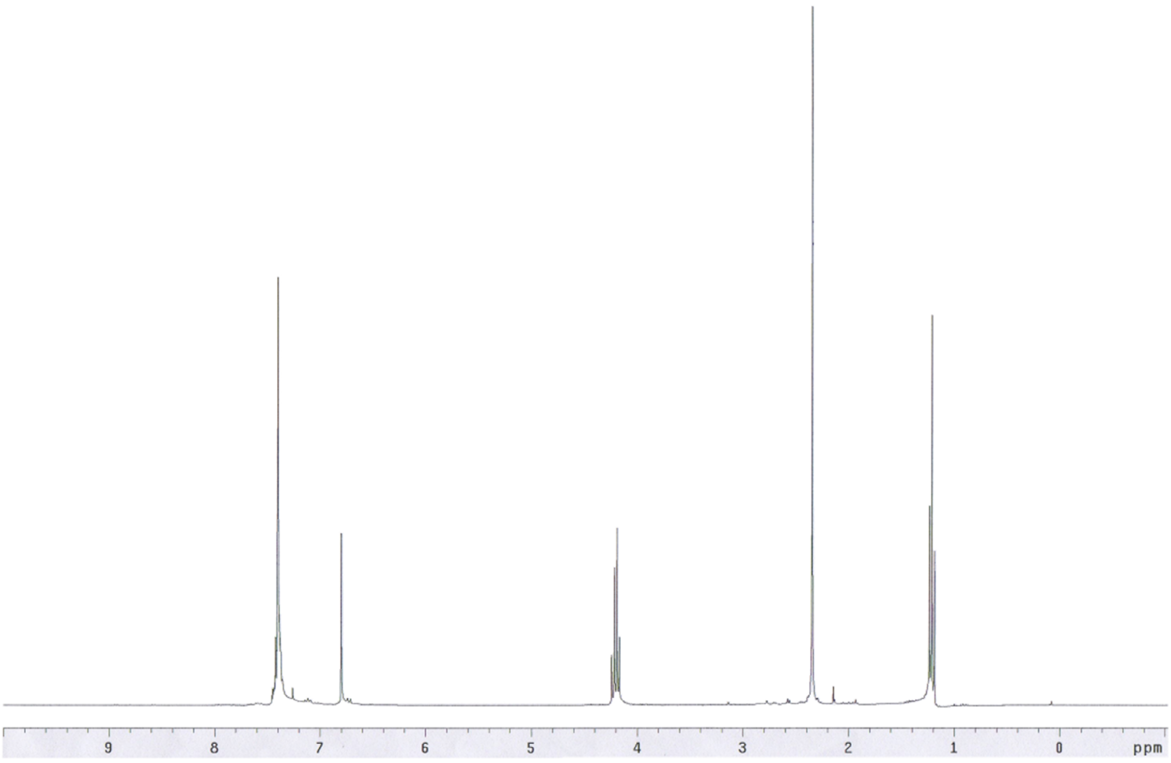

11b: Ethyl 5-methyl-1-phenyl-1*H*-pyrazole-3-carboxylate

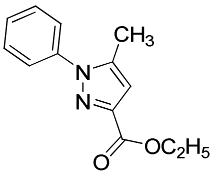

Mol. Wt.: 230.26

^{1}H NMR (500 MHz, $CDCl_3$): *δ* ppm 7.45–7.39 (m, 5H, Ar), 6.72 (s, 1H, H-4), 4.39 (q, 2H, , *J* = 7.1 Hz, -C$H_2$$CH_3$), 2.31 (s, 3H, Pyr-$CH_3$), 1.37 (t, 3H, *J* = 7.1 Hz, -CH_2CH_3). R_f = 0.48 (TLC: EP/EtOAc 8:2). Reddish-brown oil. Yield: 205 mg (35%).

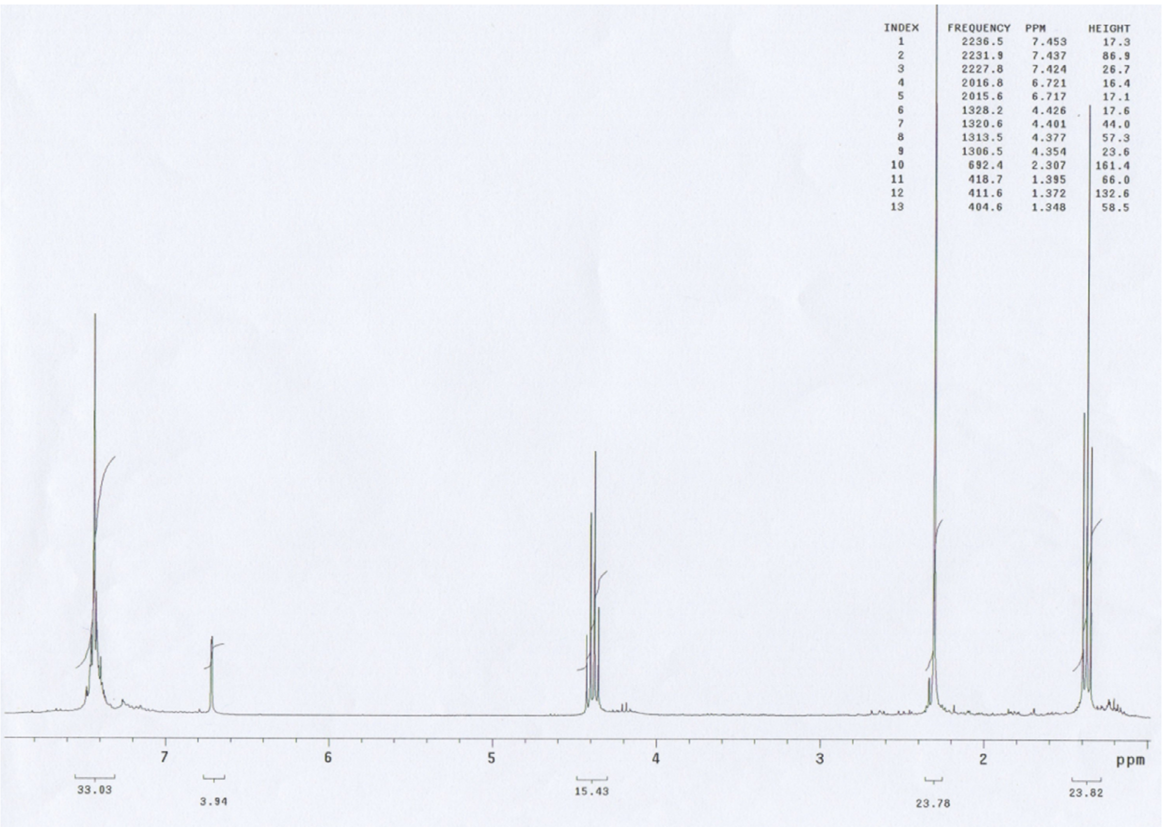

NOESY 11a:

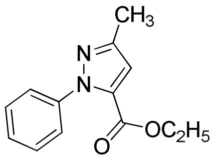

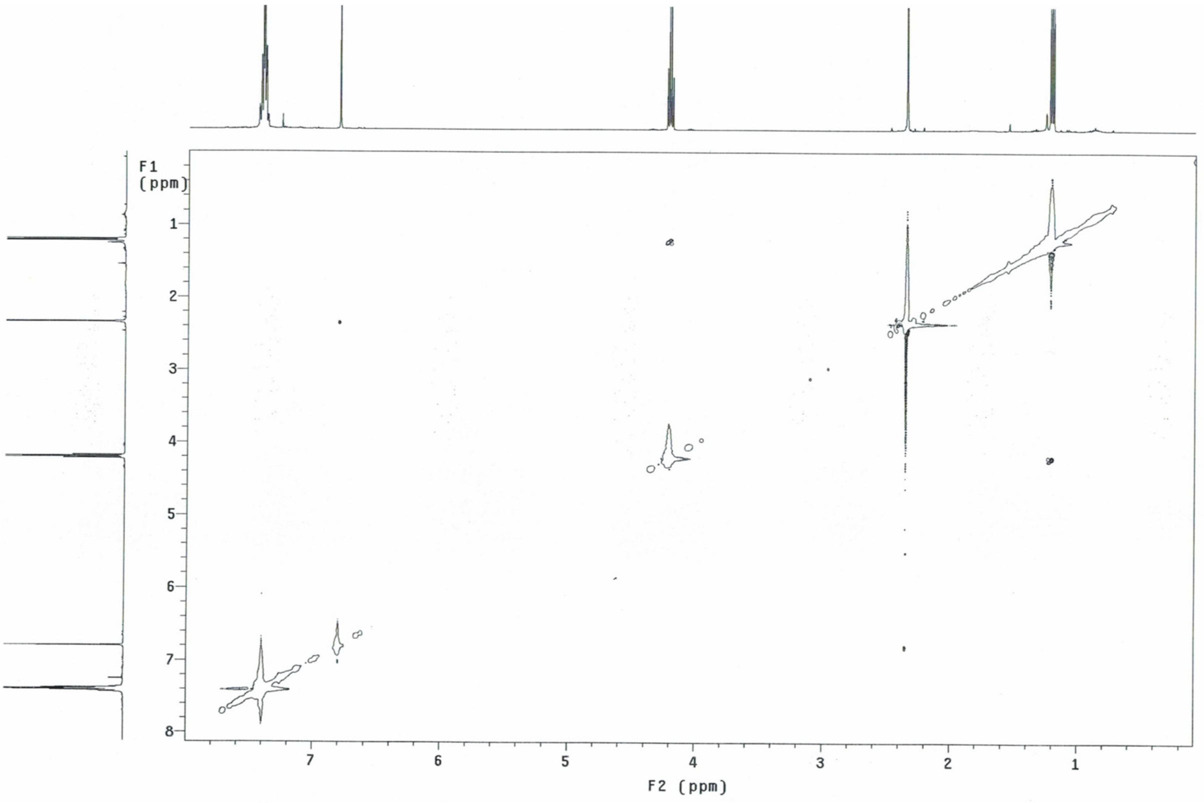

NOESY 11b:

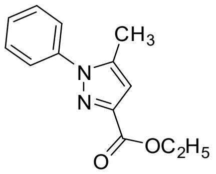

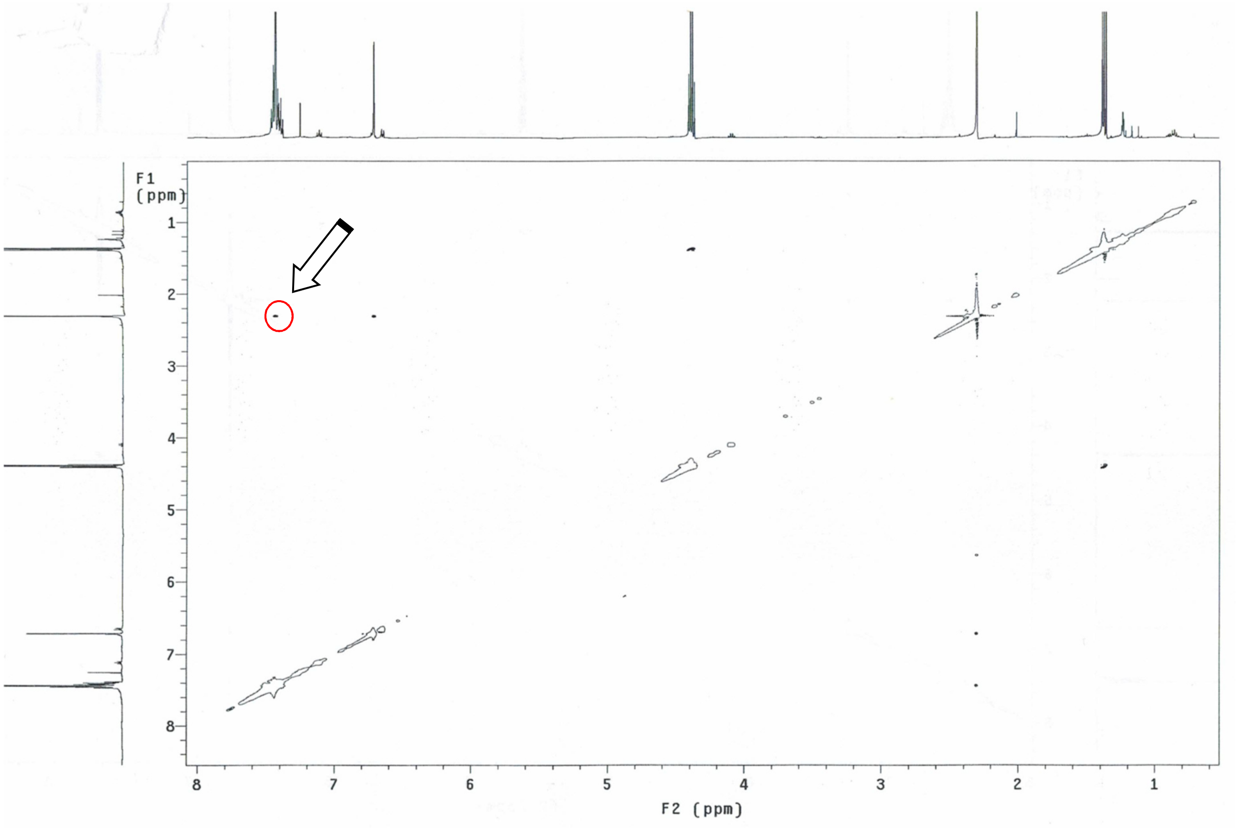

Hydrolysis of the ester moiety of the N^1-phenyl-pirazole scaffold

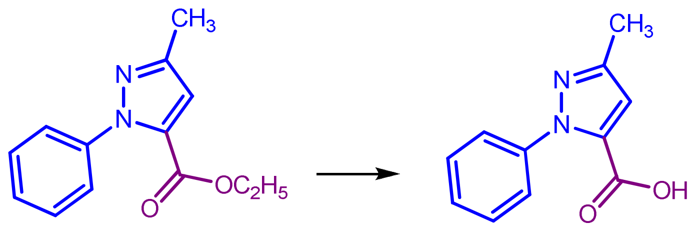

Reagents and conditions: LiOH 1 N, EtOH, 0 °C to rt, 5h.

14a: 3-Methyl-1-phenyl-1*H*-pyrazole-5-carboxylic acid

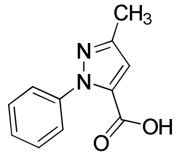

Mol. Wt.: 202.21

^{1}H NMR (500 MHz, $CDCl_3$): δ ppm 7.37–7.34 (m, 5H, Ar), 6.82 (s, 1H, H-4), 2.31 (s, 3H, CH_3). M.p. = 145–149 °C; R_f = 0.15 (TLC: 2% HCOOH in EP/EtOAc 1:1). Yellow-brownish powder. Yield: 202 mg (94%).

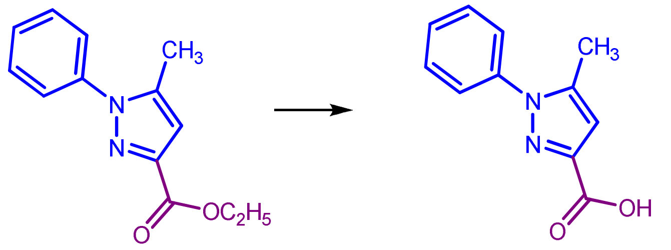

Reagents and conditions: LiOH 1 N, EtOH, 0 °C to rt, 5h.

14b: 5-Methyl-1-phenyl-1*H*-pyrazole-3-carboxylic acid

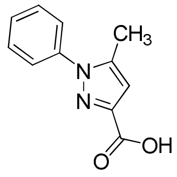

Mol. Wt.: 202.21

^{1}H NMR (500 MHz, $CDCl_3$): δ ppm 7.45–7.41 (m, 5H, Ar), 6.74 (s, 1H, H-4), 2.31 (s, 3H, CH_3). M.p. = 41–43 °C; R_f = 0.18 (TLC: 2% HCOOH in EP/EtOAc 1:1). Needle-shaped whitish crystals. Yield: 167 mg (93%).

Coupling reaction between N[1]-phenyl-pyrazole scaffold (CAP) and cinnamoyl linker

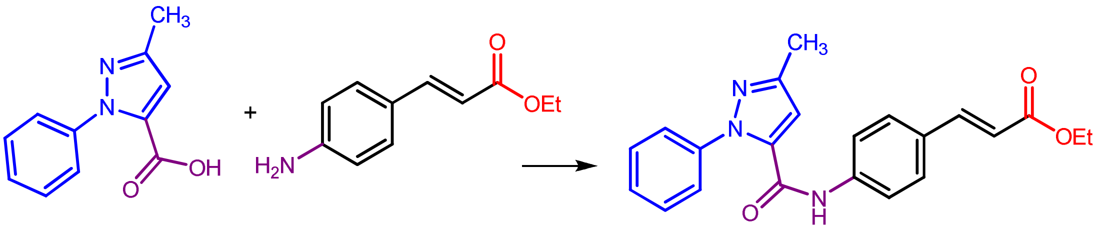

Reagents and conditions: EDCI (1.5 eq), HOBt (1.5), CH_2Cl_2, 0 °C, overnight.

17a: (*E*)-Ethyl 3-(4-(3-methyl-1-phenyl-1*H*-pyrazole-5-carboxamido)phenyl)acrylate

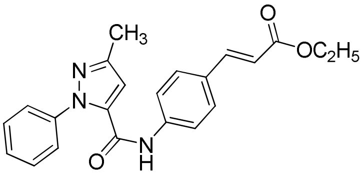

Mol. Wt.: 375.42

^{1}H NMR (500 MHz, $CDCl_3$): δ ppm 7.86 (bs, 1H, NH), 7.60 (d, *J* = 16.2 Hz, 1H, Ar-C*H*=), 7.46-7.41 (m, 9H, Ar), 6.67 (s, 1H, H-4), 6.34 (d, 1H, *J* = 16.2 Hz, =C*H*CO_2Et), 4.24 (q, *J* = 6.9 Hz, 2H, -C*H*$_2$$CH_3$), 2.35 (s, 3H, Pyr-$CH_3$), 1.32 (t, *J* = 6.9 Hz, -CH_2C*H*$_3$).

^{13}C NMR (125 MHz, $CDCl_3$): δ ppm 167.0 (-*C*OOEt), 157.4 (-*C*ONH), 149.1 (C5), 143.6 (Ar*C*H=), 139.4, 138.9, 137.3, 130.8, 129.1, 128.9, 128.6, 125.2, 119.9, 117.5 (C4), 109.3 (=CHCO), 60.5 (CH_2), 14.3 (-CH_2CH_3), 13.3 (Pyr-CH_3). R_f = 0.53 (TLC: EP/EtOAc 7:3); yellow-brownish powder. M.p. = 98–102 °C. Yield: 251 mg (67%).

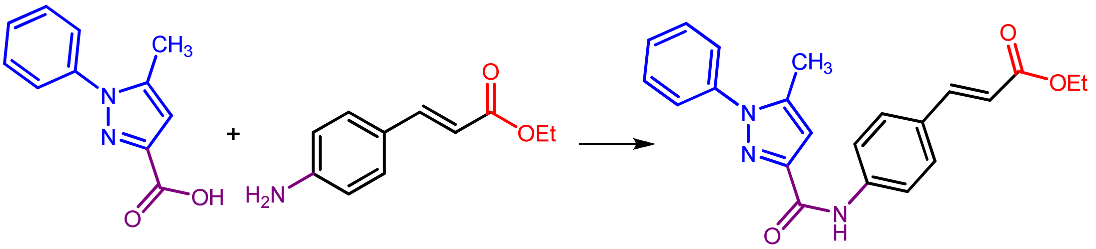

Reagents and conditions: EDCI (1.5 eq), HOBt (1.5), CH_2Cl_2, 0 °C, overnight.

17b: (*E*)-Ethyl 3-(4-(5-methyl-1-phenyl-1*H*-pyrazole-3-carboxamido)phenyl)acrylate

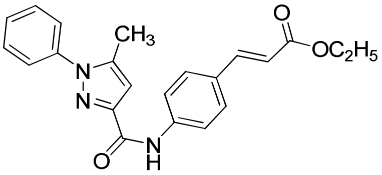

Mol. Wt.: 375.42

^{1}H NMR (500 MHz, $CDCl_3$): δ ppm 8.88 (bs, 1H, NH), 7.72 (d, 2H, *J* = 8.4, Hz, Ar*H*), 7.64 (d, *J* = 16.3 Hz, 1H, Ar-C*H*=), 7.65-7.46 (m, 7H, Ar), 6.82 (s, 1H, H-4), 6.36 (d, 1H, *J* = 16.3 Hz, =C*H*CO_2Et), 4.24 (q, *J* = 7.1 Hz, 2H, -C*H*$_2$$CH_3$), 2.35 (s, 3H, Pyr-$CH_3$), 1.32 (t, *J* = 7.1 Hz, -CH_2C*H*$_3$).

^{13}C NMR (125 MHz, $CDCl_3$): δ ppm 167.2 (-COOEt), 159.9 (-CONH), 146.5 (C3), 144.0 (ArCH=), 141.6, 139.8, 139.0, 130.0, 129.4, 129.0, 128.8, 125.2, 119.6, 116.9 (C4), 107.6 (=CHCO), 60.4 (CH_2), 14.3 (-CH_2CH_3), 12.4 (Pyr-CH_3). R_f = 0.57 (TLC: EP/EtOAc 7:3); yellowish powder. M.p. = 105–109 °C. Yield: 195 mg (63%).

Hydrolysis of the intermediate CAP-linker

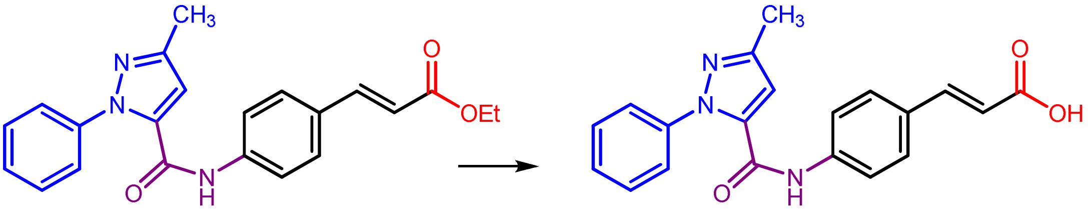

Reagents and conditions: LiOH 1 N, THF, 0 °C to rt, 6h.

20a: (*E*)-3-(4-(3-Methyl-1-phenyl-1*H*-pyrazole-5-carboxamido)phenyl)acrylic acid

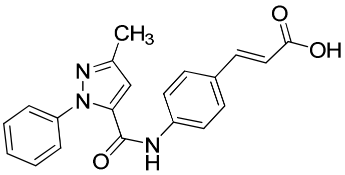

Mol. Wt.: 347.37

^{1}H NMR (500 MHz, $CDCl_3$): δ ppm 7.70 (d, *J* = 16.3 Hz, 1H, Ar-C*H*=), 7.52-7.44 (m, 10H, 9Ar*H* + N*H*), 6.71 (s, 1H, H-4), 6.37 (d, 1H, *J* = 16.3 Hz, =C*H*CO_2H), 2.39 (s, 3H, Pyr-CH_3).

^{13}C NMR (125 MHz, $CDCl_3$): δ ppm 171.5 (-COOH), 158.2 (-CONH), 148.9 (C5), 144.8 (ArCH=), 139.3, 139.1, 137.6, 131.2, 129.2, 128.9, 128.7, 125.4, 120.5, 117.7 (C4), 109.1 (=CHCO), 13.7 (Pyr-CH_3). R_f = 0.74 (TLC: 2% HCOOH in EP/EtOAc 1:1); whitish powder. M.p. = 108–110 °C. Yield: 202 mg (87%).

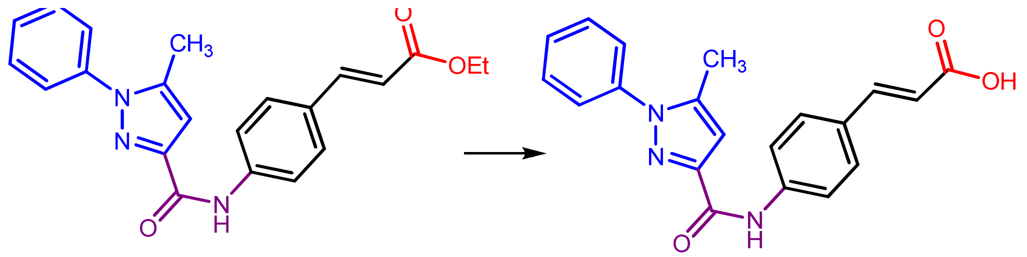

Reagents and conditions: LiOH 1 N, THF, 0°C-rt, 6h.

20b: **(*E*)-3-(4-(5-methyl-1-phenyl-1*H*-pyrazole-3-carboxamido)phenyl)acrylic acid**

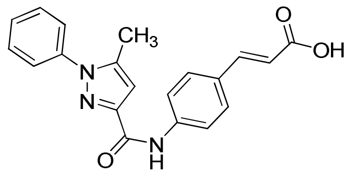

Mol. Wt.: 347.37

^{1}H NMR (500 MHz, Acetone-d_6): δ ppm 9.62 (bs, 1H, NH), 7.99 (d, 2H, *J* = 8.4 Hz, Ar*H*), 7.67 (d, 2H, *J* = 8.4 Hz, Ar*H*), 7.65-7.54 (m, 6H, 5Ar*H* + Ar-C*H*=), 6.78 (s, 1H, H-4), 6.47 (d, 1H, *J* = 15.9 Hz, =C*H*CO$_2$H), 2.41 (s, 3H, Pyr-CH$_3$).
^{13}C NMR (125 MHz, Acetone-d_6): δ ppm 167.2 (-*C*OOEt), 160.0 (-*C*ONH), 146.7 (C3), 144.0 (ArCH=), 141.6, 139.5, 129.8, 129.3, 128.9, 128.5, 127.0, 125.2, 119.7, 117.0 (C4), 107.3 (=CHCO), 11.5 (Pyr-CH$_3$). R_f = 0.41 (TLC: 2% HCOOH in EP/EtOAc 1:1); whitish powder. M.p. = 143–147 °C. Yield: 123 mg (68%).

Synthesis of the final hydroxamic acid with CAP N*[1]*-phenyl-pyrazole (1a)

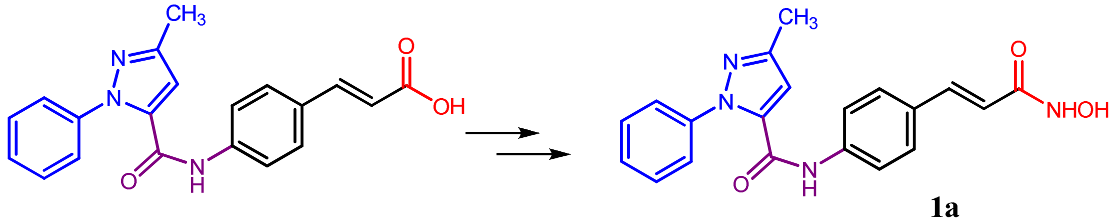

1a

Reagents and conditions: (1) TBDMSiO-NH$_2$ (1 eq), EDCI (1.5 eq), CH$_2$Cl$_2$, 0 °C to r.t., overnight; (2) TFA, CH$_2$Cl$_2$, 0 °C, 5h.

1a: **(*E*)-N-(4-(3-(Hydroxyamino)-3-oxoprop-1-enyl)phenyl)-3-methyl-1-phenyl-1*H*-pyrazole-5-carboxamide**

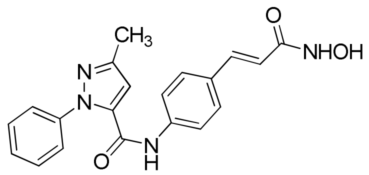

Mol. Wt.: 362.38

^{1}H NMR (500 MHz, CD_3OD): *δ* ppm 7.63–7.40 (m, 10H, 9Ar*H* + Ar-C*H*=), 6.79 (s, 1H, H-4), 6.40 (d, 1H, *J* = 15.9 Hz, =C*H*CONHOH), 2.36 (s, 3H, Pyr-CH_3).

^{13}C NMR (125 MHz, CD_3OD): *δ* ppm 165.0 (-CONHOH), 158.9 (-CONH), 149.1 (C5), 139.9 (ArCH=), 139.4, 137.9, 131.1, 128.6, 128.0, 127.8, 126.1, 124.5, 120.2, 116.2 (C4), 108.6 (=CHCO), 13.2 (Pyr-CH_3). R_f = 0.51 (TLC: EtOAc/MeOH 8:2); yellowish powder. M.p. = 103–107 °C. Yield: 101 mg (48%). Anal. Calcd for $C_{20}H_{18}N_4O_3$: C, 66.29; H, 5.01; N, 15.46. Found: C, 66.35; H, 5.03; N, 15.50.

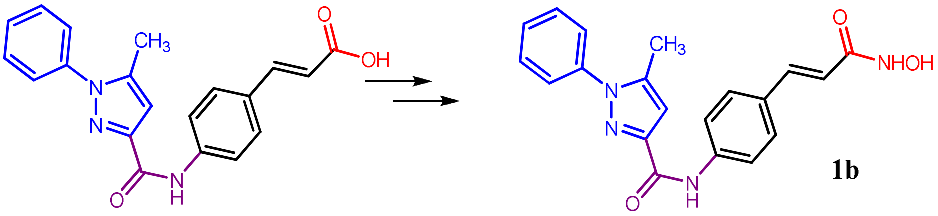

1b

Reagents and conditions: (1) TBDMSiO-NH_2 (1 eq), EDCI (1.5 eq), CH_2Cl_2, 0 °C to r.t., overnight; (2) TFA, CH_2Cl_2, 0 °C, 5h.

1b: (*E*)-N-(4-(3-(Hydroxyamino)-3-oxoprop-1-enyl)phenyl)-5-methyl-1-phenyl-1*H*-pyrazole-3-carboxamide

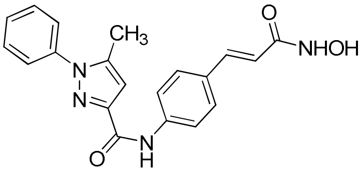

Mol. Wt.: 362.38

^{1}H NMR (500 MHz, CD_3OD): *δ* ppm 7.81 (d, 2H, *J* = 8.4 Hz, Ar*H*), 7.72 (d, 2H, *J* = 8.4 Hz, Ar*H*), 7.65-7.54 (m, 5H, Ar*H*), 7.53 (d, 1H, *J* = 13.8 Hz, Ar-C*H*=), 6.80 (s, 1H, H-4), 6.30 (d, 1H, *J* = 13.8 Hz, =C*H*CONHOH), 2.36 (s, 3H, Pyr-CH_3).

^{13}C NMR (125 MHz, CD_3OD): *δ* ppm 165.2 (-CONHOH), 159.8 (-CONH), 148.1 (C3), 143.4 (ArCH=), 140.7, 139.6, 129.9, 129.3, 129.1, 128.4, 126.8, 125.4, 118.8, 117.1 (C4), 107.5 (=CHCO), 11.9 (Pyr-CH_3). R_f = 0.18 (TLC: EtOAc/MeOH 8:2); yellowish powder. M.p. = 103–107 °C. Yield: 45 mg (42%). Anal. Calcd for $C_{20}H_{18}N_4O_3$: C, 66.29; H, 5.01; N, 15.46. Found: C, 66.26; H, 4.99; N, 15.49.

***Synthesis of the other hydroxamic acids with CAP N*[1]*-aryl-pyrazole* (2-3)**

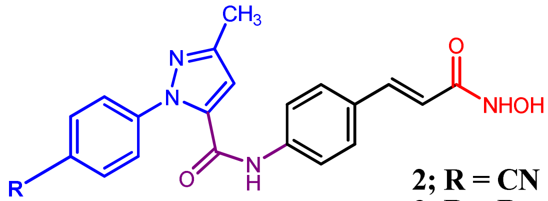

2; R = CN
3; R = Br

In regard to the other N^1-aryl-pyrazole derivatives (*i.e.* *p*-cyanophenyl **2** and *p*-bromophenyl **3**), the design and synthesis were carried out only on the related pyrazole scaffolds of the isomers **12a** and **13a**, respectively, in accordance with the preliminary biological data obtained with the N^1-phenyl analogs **1a**,**b**.

Compound 2

Ethyl 1-(4-cyanophenyl)-3-methyl-1*H*-pyrazole-5-carboxylate (12a)

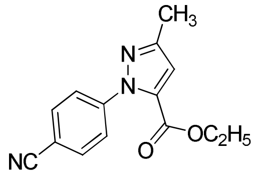

Mol. Wt.: 255.27

^{1}H NMR (500 MHz, $CDCl_3$): δ ppm 7.71 (d, 2H, *J* = 8.8 Hz, H-2′,6′), 7.55 (d, 2H, *J* = 8.8 Hz, H-3′,5′), 6.85 (s, 1H, H-4), 4.25 (q, 2H, *J* = 7.4 Hz, -CH_2CH_3), 2.34 (s, 3H, Pyr-CH_3), 1.28 (t, 3H, *J* = 7.4 Hz, -CH_2CH_3). R_f = 0.66 (TLC: EP/EtOAc 7:3). M.p. = 154–155 °C. White solid.

1-(4-Cyanophenyl)-3-methyl-1*H*-pyrazole-5-carboxylic acid (15a)

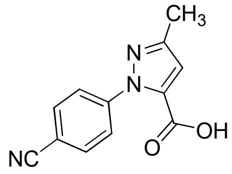

Mol. Wt.: 227.22

^{1}H NMR (500 MHz, Acetone-d_6): δ ppm 7.90 (d, 2H, *J* = 8.3 Hz, H-2′,6′), 7.73 (d, 2H, *J* = 8.3 Hz, H-3′,5′), 6.94 (s, 1H, Pyr-H-4), 2.31 (s, 3H, CH_3). White powder. R_f = 0.26 (TLC: 2% HCOOH in EP/EtOAc 1:1). M.p. = 225–226 °C.

(*E*)-ethyl 3-(4-(1-(4-cyanophenyl)-3-methyl-1*H*-pyrazole-5-carboxamido)phenyl)acrylate (18a)

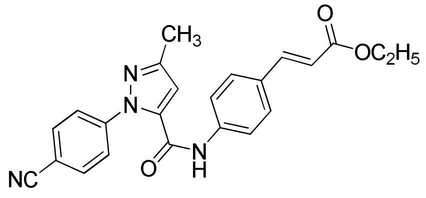

Mol. Wt.: 400.43

^{1}H NMR (500 MHz, $CDCl_3$): δ 8.58 (bs, 1H, NH), ppm 7.64 (d, 2H, *J* = 8.3 Hz, Ar*H*), 7.60 – 7.55 (m, 5H, 4Ar*H* + ArC*H*=), 7.44 (d, 2H, *J* = 8.8 Hz, Ar), 6.67 (s, 1H, Pyr-H-4), 6.34 (d, 1H, *J* = 15.7 Hz, =C*H*CO), 4.23 (q, 2H, *J* = 7.3 Hz, -CH_2CH_3), 2.31 (s, 3H,Pyr-CH_3), 1.31 (t, 3H, *J* = 7.3 Hz, -CH_2CH_3).

^{13}C NMR (125 MHz, $CDCl_3$): δ ppm 167.1 (-*C*OOEt), 157.5 (-*C*ONH), 150.1 (C5), 143.5 (ArCH=), 143.1, 138.9, 137.0, 132.6, 130.9, 128.9, 125.0, 120.1, 118.2 (-CN), 117.5 (C4), 111.0 (*C*-CN), 110.0 (=CHCO), 60.5 (CH_2), 14.2 (-CH_2CH_3), 13.3 (Pyr-CH_3). White powder. R_f = 0.38 (TLC: EP/EtOAc 6:4). M.p. = 184–186 °C.

(*E*)-3-(4-(1-(4-cyanophenyl)-3-methyl-1*H*-pyrazole-5-carboxamido)phenyl)acrylic acid (21a)

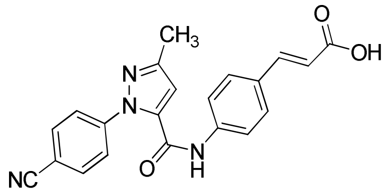

Mol. Wt.: 372.38

^{1}H NMR (500 MHz, Acetone-*d*6): δ ppm 9.99 (bs, 1H, NH), 7.88 (d, 2H, *J* = 8.8 Hz, Ar*H*), 7.81 (d, 2H, *J* = 8.3 Hz, Ar*H*), 7.75 (d, 2H, *J* = 8.3 Hz, Ar*H*), 7.68 (d, 2H, *J* = 8.8 Hz, Ar*H*), 7.64 (d, 1H, *J* = 15.7 Hz, ArC*H*=), 6.93 (s, 1H, Pyr-H-4), 6.47 (d, 1H, *J* = 15.7 Hz, =C*H*CO), 2.32 (s, 3H,Pyr-CH_3).

^{13}C NMR (125 MHz, Acetone-*d*6): δ ppm 173.9 (-*C*OOH), 159.4 (-*C*ONH), 149.1 (C5), 143.8 (ArCH=), 133.7, 132.6, 128.8, 128.3, 124.9, 120.21, (-CN), 116.7 (C4), 108.5 (*C*-CN), 103.3 (=CHCO), 13.3 (Pyr-CH_3). White powder. R_f = 0.43 (TLC: 2% HCOOH in EP/EtOAc 6:4); M.p. = 248–250 °C.

(*E*)-1-(4-cyanophenyl)-*N*-(4-(3-(hydroxyamino)-3-oxoprop-1-enyl)phenyl)-3-methyl-1*H*-pyrazole-5-carboxamide (2)

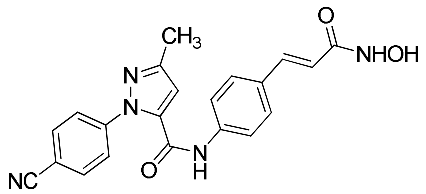

Mol. Wt.: 387.39

^{1}H NMR (500 MHz, CD_3OD): δ ppm 8.00–7.38 (m, 9H, 8Ar*H* + Ar-C*H*=), 6.90 (s, 1H, H-4), 6.83 (d, 1H, *J* = 15.9 Hz, =C*H*CONHOH), 2.35 (s, 3H, Pyr-CH_3).

^{13}C NMR (125 MHz, CD_3OD): δ ppm 164.9 (-*C*ONHOH), 158.7 (-*C*ONH), 150.3 (C5), 143.3 (ArCH=), 132.7, 129.2, 128.9, 128.1, 126.4, 124.9, 124.1 120.3 (-CN), 117.8 (C4), 115.1 (*C*-CN), 110.6 (=CHCO), 11.2 (Pyr-CH_3). Yellowish powder. R_f = 0.12 (TLC: EtOAc/MeOH 8:2); M.p. = 110–114 °C. Anal. Calcd for $C_{21}H_{17}N_5O_3$: C, 65.11; H, 4.42; N, 18.08. Found: C, 65.18; H, 4.45; N, 18.13.

Compound 3

Ethyl 1-(4-bromophenyl)-3-methyl-1*H*-pyrazole-5-carboxylate (13a)

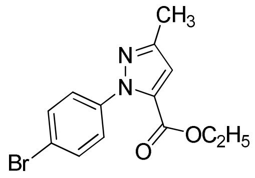

Mol. Wt.: 309.16

^{1}H NMR (500 MHz, $CDCl_3$): δ ppm 7.56 (d, 2H, *J* = 8.8 Hz, H-3′,5′), 7.39 (d, 2H, *J* = 8.8 Hz, H-2′,6′), 6.81 (s, 1H, H-4), 4.24 (q, 2H, *J* = 7.3 Hz, -C*H*$_2$CH$_3$), 2.35 (s, 3H, Pyr-CH$_3$), 1.26 (t, 3H, *J* = 7.3 Hz, -CH$_2$C*H*$_3$). R_f = 0.75 (TLC: EP/EE 7:3). Whitish needles. M.p. = 137–138 °C.

1-(4-Bromophenyl)-3-methyl-1*H*-pyrazole-5-carboxylic acid (16a)

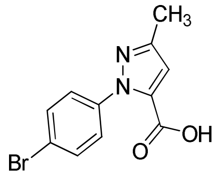

Mol. Wt.: 281.11

^{1}H NMR (300 MHz, $CDCl_3$): δ ppm 7.55 (d, 2H, *J* = 8.8 Hz, H-3′,5′), 7.29 (d, 2H, *J* = 8.8 Hz, H-2′,6′), 6.87 (s, 1H, H-4), 2.36 (s, 3H, Pyr-CH$_3$). White solid. R_f = 0.29 (TLC: 2% HCOOH in EP/EtOAc 1:1); M.p. = 211–213 °C.

(*E*)-Ethyl 3-(4-(1-(4-bromophenyl)-3-methyl-1*H*-pyrazole-5-carboxamido)phenyl)acrylate (19a)

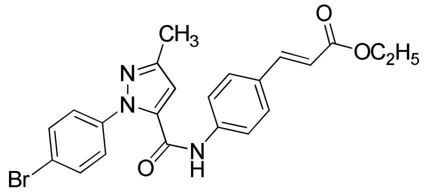

Mol. Wt.: 454.32

^{1}H NMR (300 MHz, $CDCl_3$): δ ppm 7.87 (bs, 1H, NH), 7.61 (d, 2H, *J* = 8.4 Hz, Ar*H*), 7.52 (d, 2H, *J* = 8.4 Hz, Ar*H*), 7.46 (d, 2H, *J* = 8.4 Hz, Ar*H*), 7.42 (d, 1H, ArC*H*=), 7.31 (d, 2H, *J* = 8.4 Hz, Ar*H*), 6.62 (s, 1H, Pyr-H-4), 6.35 (d, 1H, *J* = 15.7 Hz, =C*H*CO), 4.24 (q, 2H, *J* = 7.3 Hz, -C*H*$_2$CH$_3$), 2.34 (s, 3H,Pyr-CH$_3$), 1.32 (t, 3H, *J* = 7.3 Hz, -CH$_2$C*H*$_3$).

^{13}C NMR (75 MHz, $CDCl_3$): δ ppm 168.6 (-*C*OOEt), 159.5 (-CONH), 148.8 (C5), 144.2 (Ar*C*H=), 141.1, 139.5, 137.7, 132.6, 130.0, 128.9, 126.6, 121.3 (C-Br), 118.9, 117.1 (C4), 106.6 (=CHCO), 61.0 (CH$_2$), 13.4 (-CH$_2$CH$_3$), 12.3 (Pyr-*C*H$_3$). Whitish powder. R_f = 0.75 (TLC: EP/EtOAc 6:4); M.p. = 181–183 °C.

(*E*)-3-(4-(1-(4-Bromophenyl)-3-methyl-1*H*-pyrazole-5-carboxamido)phenyl)acrylic acid (22a)

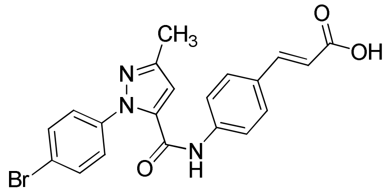

Mol. Wt.: 426.26

^{1}H NMR (300 MHz, Acetone-d_6): δ ppm 9.96 (bs, 1H, NH), 7.90 (d, 2H, *J* = 8.3 Hz, Ar*H*), 7.77–7.70 (m, 5H, 4Ar*H* + ArC*H*=), 7.56 (d, 2H, *J* = 8.3 Hz, Ar*H*), 6.95 (s, 1H, Pyr-H-4), 6.54 (d, 1H, *J* = 15.7 Hz, =C*H*CO), 2.38 (s, 3H,Pyr-CH_3).

^{13}C NMR (75 MHz, $CDCl_3$): δ ppm 167.0 (-*C*OOH), 158.0 (-*C*ONH), 148.7 (C5), 144.1 (Ar*C*H=), 140.8, 140.5, 137.8, 131.5, 130.0, 128.9, 126.5, 121.3 (C-Br), 120.0, 117.3 (C4), 109.5 (=*C*HCO), 12.6 (Pyr-CH_3). Whitish powder. R_f = 0.23 (TLC: 2% HCOOH in EP/EtOAc 6:4); M.p. = 238–241 °C.

(*E*)-1-(4-Bromophenyl)-*N*-(4-(3-(hydroxyamino)-3-oxoprop-1-enyl)phenyl)-3-methyl-1*H*-pyrazole-5-carboxamide (3)

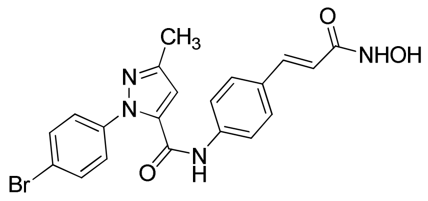

Mol. Wt.: 441.28

^{1}H NMR (300 MHz, CD_3OD): δ ppm 7.83 – 7.22 (m, 9H, 8Ar*H* + Ar-C*H*=), 6.83 (s, 1H, H-4), 6.40 (d, 1H, *J* = 15.9 Hz, =C*H*CONHOH), 2.35 (s, 3H, Pyr-CH_3).

^{13}C NMR (75 MHz, $CDCl_3$): δ ppm 166.1 (-CONHOH), 158.7 (-CONH), 148.9 (C5), 143.4 (ArCH=), 140.7, 139.7, 137.8, 131.6, 130.5, 128.7, 126.1, 122.0 (C-Br), 119.8, 117.0 (C4), 109.2 (-CHCO), 13.0 (Pyr-CH_3). R_f = 0.13 (TLC: EtOAc/MeOH 8:2); M.p. = 103–107 °C. Pale pink solid. Anal. Calcd for $C_{20}H_{17}BrN_4O_3$: C, 54.44; H, 3.88; N, 12.70. Found: C, 54.52; H, 3.89; N, 12.73.

General procedure for the synthesis of N¹H-pyrazole derivatives

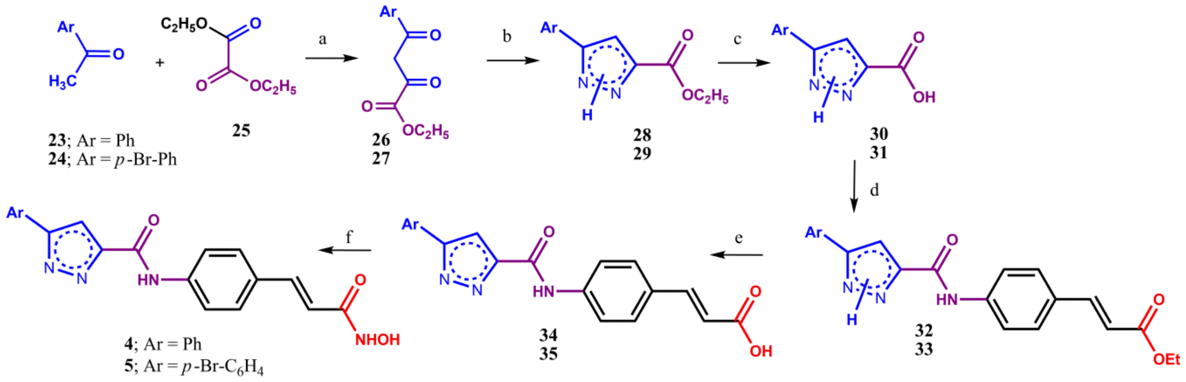

Scheme S2. Reagents and conditions a) EtONa, EtOH, Δ, 4h; b) N_2H_4, EtOH, Δ, 3h; c) LiOH 1N, EtOH, 0 °C to r.t., 5h; d) EDCI, HOBt, CH_2Cl_2, 0 °C to r.t., overnight; e) LiOH 1N, EtOH, 0 °C to r.t., 6h; f) TBDMSiO-NH_2, EDCI, CH_2Cl_2, 0 °C to r.t., overnight; then TFA, CH_2Cl_2, 0 °C, 5h.

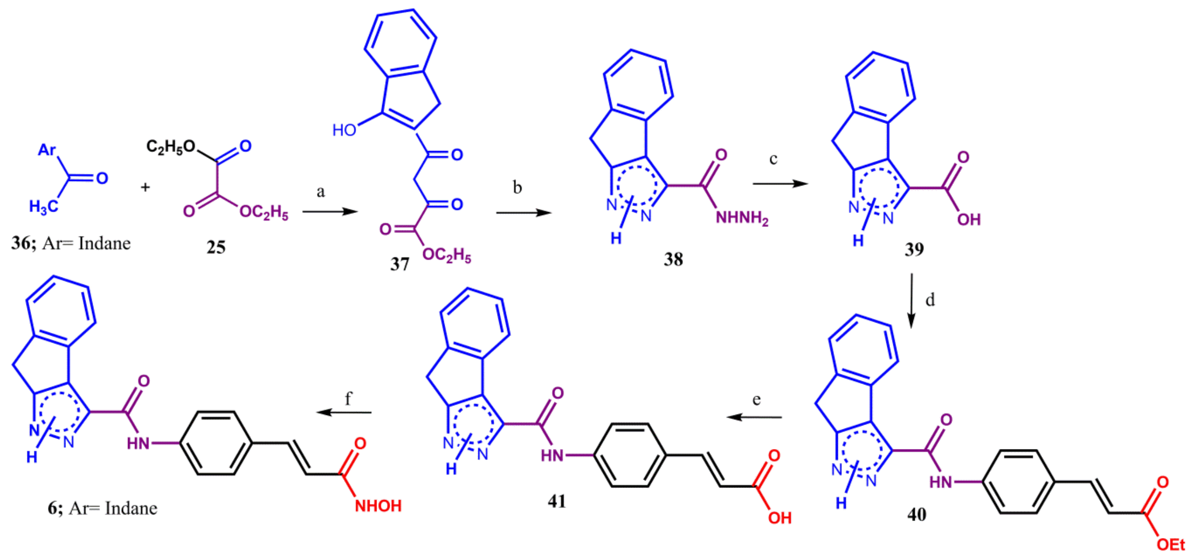

Scheme S3. Reagents and conditions a) EtONa, EtOH, Δ, 4h; b) N_2H_4, EtOH, Δ, 3h; c) HCl/CH_3COOH_{glac} 1:1, overnight; d) EDCI, HOBt, CH_2Cl_2, 0 °C to r.t., overnight; e) LiOH 1N, EtOH, 0 °C to r.t., 6h; f) TBDMSiO-NH_2, EDCI, CH_2Cl_2, 0 °C to r.t., overnight; then TFA, CH_2Cl_2, 0 °C, 5h.

Synthesis of the α,γ-diketo-ester intermediate of the phenyl derivative

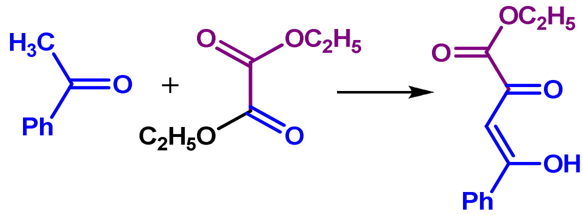

Reagents and conditions: EtONa, EtOH, Δ, 4h.

(*Z*)-Ethyl 4-hydroxy-2-oxo-4-phenylbut-3-enoate (26)

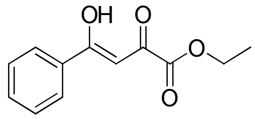

Mol. Wt.: 220.22

^{1}H NMR (500 MHz, $CDCl_3$): δ ppm 7.98 (d, 2H, *J* = 7.5 Hz, Ph*H*-2′-6′), 7.59 (t, 1H, *J* = 7.5 Hz, Ph*H*-4′), 7.49 (t, 2H, *J* = 7.5 Hz, Ph*H*-3′-5′), 7.07 (s, 1H, -CH=), 4.39 (q, 2H, *J* = 7.1 Hz, -C*H*$_2$CH$_3$), 1.40 (t, 3H, *J* = 7.1 Hz, -CH$_2$C*H*$_3$). R_f = 0.76 (TLC: CH_2Cl_2/CH_3OH 95:5). Dark brown sticky oil. Resa: 1.17 g (85%).

Synthesis of N*[1]*H-pyrazole scaffold C5-phenyl-substituted

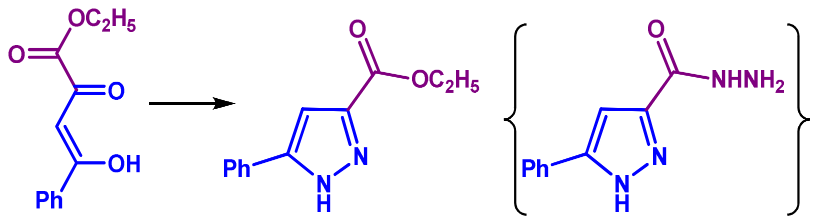

Reagents and conditions: N_2H_4, EtOH, Δ, 3h.

5-Phenyl-1*H*-pyrazole-3-carbohydrazide

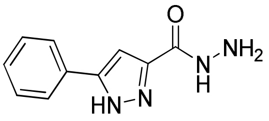

Mol. Wt.: 202.21

^{1}H NMR (500 MHz, DMSO-d_6): δ ppm 9.55 (bs, 1H, CON*H*-), 7.74 (d, 2H, *J* = 7.4 Hz, Ph*H*-2′-6′), 7.43 (t, 2H, *J* = 7.4 Hz, Ph*H*-3′,5′), 7.32 (t, 1H, *J* = 7.4 Hz, Ph*H*-4′), 7.10 (s, 1H, Pyr*H*-4), 4.44 (bs, 2H, -NH_2). R_f = 0.10 (TLC: 1% HCOOH in CH_2Cl_2/CH_3OH 95:5). Old rose powder; M.p. = 95–98 °C. Yield: 537 mg (50%).

(*Z*)-Ethyl 4-hydroxy-2-oxo-4-phenylbut-3-enoate (28)

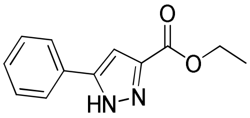

Mol. Wt.: 216.24

^{1}H NMR (500 MHz, $CDCl_3$): δ ppm 7.75 (d, 2H, *J* = 7.9 Hz, Ph*H*-2′-6′), 7.42 (t, 2H, *J* = 7.9 Hz, Ph*H*-3′-5′,) 7.35 (t, 1H, *J* = 7.9 Hz, Ph*H*-4′), 7.09 (s, 1H, Pyr*H*), 4.36 (q, 2H, *J* = 7.1 Hz, -C*H*$_2$CH$_3$), 1.36 (t, 3H, *J* = 7.1 Hz, -CH$_2$C*H*$_3$). ^{13}C NMR (125 MHz, $CDCl_3$): δ ppm 160.5 (-COOEt), 149.4 (C5), 140.2 (C3), 130.7, 128.9, 128.6, 125.7, 105.5 (C4), 61.3 (CH_2), 14.2 (-CH_2CH_3). R_f = 0.66 (TLC: *n*-Hexane/EtOAc 7:3). Whitish solid; M.p. = 65–68 °C. Yield: 437 mg (38%).

Hydrolysis of the N[1]H-pyrazole scaffolds C5-phenyl-substituted

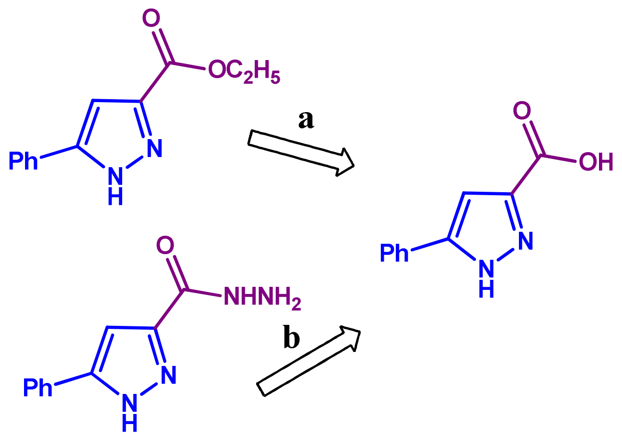

Reagents and conditions: a) LiOH 1N, EtOH, 0 °C to r.t., 5h; b) HCl 6N/$CH_3COOH_{gl.}$ (1:1), Δ, overnight.

Ethyl 5-phenyl-1*H*-pyrazole-3-carboxylate (30)

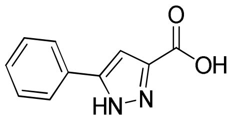

Mol. Wt.: 118.18

^{1}H NMR (500 MHz, DMSO-d_6): δ ppm 13.78 (bs, 1H, PyrN*H*), 13.02 (bs, 1H, -COO*H*), 7.81 (d, 2H, *J* = 7.4 Hz, Ph*H*-2′-6′), 7.42 (t, 2H, *J* = 7.4 Hz, Ph*H*-3′,5′), 7.33 (t, 1H, *J* = 6.9 Hz, Ph*H*-4′), 7.17 (s, 1H, Pyr*H*-4). R_f = 0.13 (TLC: 1% HCOOH in CH_2Cl_2/CH_3OH 95:5). Whitish powder; M.p. = 119–121 °C. Yield route a: 350 mg (92%); yield route b: 426 mg (85%).

Coupling reaction between N¹H-5-phenyl-pyrazole scaffold (CAP) and cinnamoyl linker

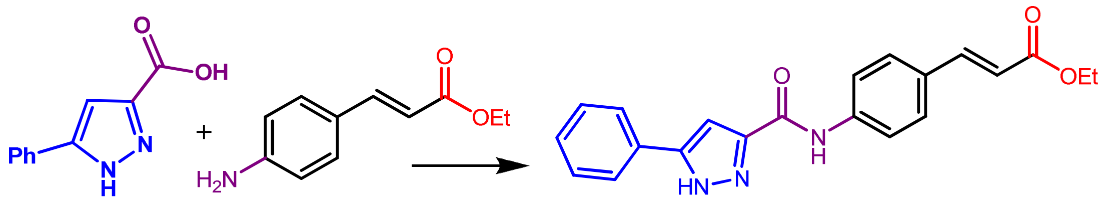

Reagents and conditions: EDCI (1.5 eq), HOBt (1.5 eq), CH_2Cl_2, 0 °C to r.t., overnight.

(*E*)-Ethyl 3-(4-(5-phenyl-1*H*-pyrazole-3-carboxamido)phenyl)acrylate (32)

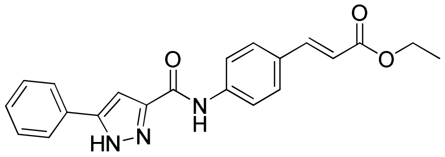

Mol. Wt.: 361.39

^{1}H NMR (500 MHz, $CDCl_3$): δ ppm 8.99 (bs, 1H, -CON*H*), 7.76 (d, 2H, *J* = 8.8 Hz, Ar*H*), 7.70 (d, 1H, *J* = 15.9 Hz, ArC*H*=), 7.64 (d, 2H, *J* = 8.8 Hz, Ar*H*), 7.54 (d, 2H, *J* = 8.4 Hz, Ar*H*), 7.48 (t, 2H, *J* = 8.4 Hz, Ar*H*), 7.43 (t, 1H, *J* = 8.4 Hz, Ar*H*), 7.19 (s, 1H, Pyr*H*-4), 6.40 (d, 1H, *J* = 15.9 Hz, =C*H*CO), 4.27 (q, 2H, *J* = 7.0 Hz, -C*H*$_2$CH$_3$), 1.34 (t, 3H, *J* = 7.0 Hz, -CH$_2$C*H*$_3$).

^{13}C NMR (125 MHz, $CDCl_3$): δ ppm 170.1 (-COOEt), 160.7 (-CONH), 148.2 (C3), 143.8 (ArCH=), 140.9 (C5), 138.7, 131.9, 130.1, 129.4, 128.4, 125.6, 121.3, 118.1, 117.2 (=CHCO), 103.8 (C4), 61.2 (CH_2), 13.1 (-CH_2CH_3). R_f = 0.57 (TLC: CH_2Cl_2/EtOAc 8:2). Pale yellow powder; M.p. = 142–145 °C. Yield: 383 mg (57%).

Hydrolysis of the adduct N¹H-pyrazole CAP/cinnamoyl linker

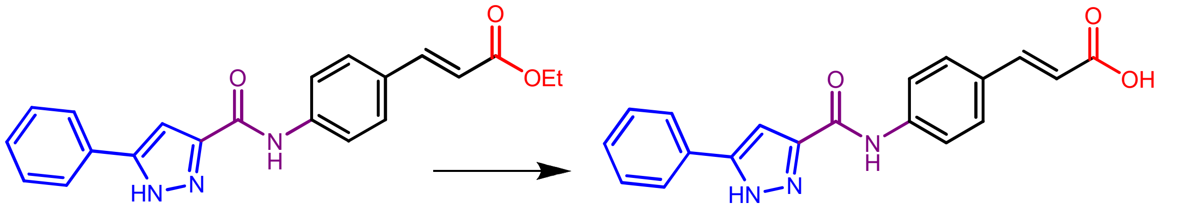

Reagents and conditions: LiOH 1N, EtOH, 0 °C to r.t., 6h.

(*E*)-3-(4-(5-Phenyl-1*H*-pyrazole-3-carboxamido)phenyl)acrylic acid (34)

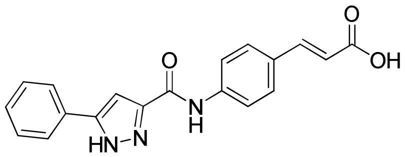

Mol. Wt.: 333.34

^{1}H NMR (500 MHz, DMSO-*d*$_6$): *δ* ppm 13.83 (bs, 1H, PyrN*H*), 12.26 (bs, 1H, -COO*H*), 10.30 (bs, 1H, -CON*H*), 7.89 (d, 2H, *J* = 8.0 Hz, Ar*H*), 7.83 (d, 2H, *J* = 7.5 Hz, Ar*H*), 7.66 (d, 2H, *J* = 8.0 Hz, Ar*H*), 7.54 (d, 1H, *J* = 15.9 Hz, ArC*H*=), 7.47 (t, 2H, *J* = 7.5 Hz, Ar*H*), 7.37 (t, 1H, *J* = 7.5 Hz, Ar*H*), 7.45 (s, 1H, Pyr*H*-4), 6.44 (d, 1H, *J* = 15.9 Hz, =C*H*CO).
^{13}C NMR (125 MHz, DMSO-*d*$_6$): *δ* ppm 168.2 (-COOH), 161.5 (-CONH), 148.1 (C3), 144.1 (ArCH=), 141.1 (C5), 138.8, 131.7, 130.0, 129.5, 128.2, 125.8, 121.5, 118.2, 117.9 (=CHCO), 103.7 (C4). R$_f$ = 0.15 (TLC: 2% HCOOH in CH_2Cl_2/MeOH 95:5). Beige powder; M.p. = 171–174 °C. Yield: 219 mg (62%).

Synthesis of the final hydroxamic acid with CAP N[1]H-5-phenyl-pyrazole (4)

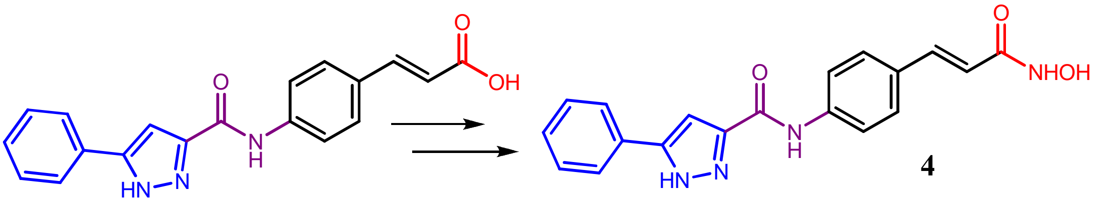

Reagents and conditions: (1) TBDMSiO-NH_2 (1 eq), EDCI (1.5 eq), CH_2Cl_2, 0 °C to r.t., overnight; (2) TFA, CH_2Cl_2, 0 °C, 5h.

(*E*)-*N*-(4-(3-(Hydroxyamino)-3-oxoprop-1-enyl)phenyl)-5-phenyl-1*H*-pyrazole-3-carboxamide (4)

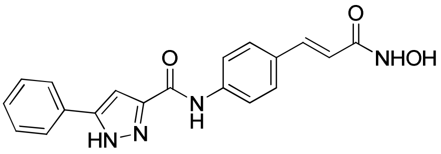

Mol. Wt.: 348.36
^{1}H NMR (500 MHz, DMSO-*d*$_6$): *δ* ppm 13.89 (bs, 1H, PyrN*H*), 9.64 (bs, 1H, -CON*H*), 7.84 (d, 2H, *J* = 8.8 Hz, Ar*H*), 7.75 (d, 2H, *J* = 7.9 Hz, Ar*H*), 7.73 (d, 1H, *J* = 16.1 Hz, ArC*H*=), 7.64 (d, 2H, *J* = 8.8 Hz, Ar*H*), 7.47 (t, 2H, *J* = 7.9 Hz, Ar*H*), 7.38 (t, 1H, *J* = 7.9 Hz, Ar*H*), 7.20 (s, 1H, Pyr*H*-4), 6.62 (d, 1H, *J* = 16.1 Hz, =C*H*CO).
^{13}C NMR (125 MHz, DMSO-*d*$_6$): *δ* ppm 165.5 (-CONHOH), 161.0 (-CONH), 158.9 (ArCH=), 147.2 (C3), 141.1 (C5), 137.9, 131.3, 130.0, 129.5, 129.0, 125.9, 120.9, (=CHCO), 103.7 (C4). R$_f$ = 0.14 (TLC: 2% HCOOH in EtOAc/MeOH 8:2). Yellowish powder; M.p. = 254–258 °C. Yield: 87 mg (38%). Anal. Calcd for $C_{19}H_{16}N_4O_3$: C, 65.51; H, 4.63; N, 16.08. Found: C, 65.59; H, 4.60; N, 16.12.

Synthesis of the other hydroxamic acids with CAP N[1]H-aryl-substituted-pyrazole (5-6)

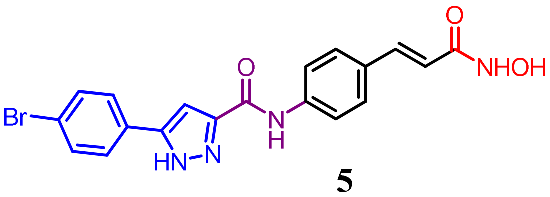

5

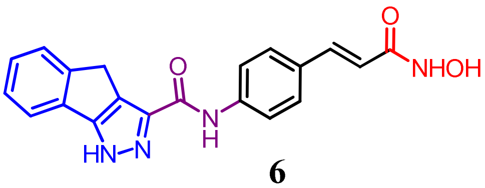

6

In regard to this second group of derivatives, the other compounds, which were designed and synthetized with the same procedure employed for the phenyl-derivative **4**, are the *p*-bromophenyl (**5**) and the 1-indanone derivative (**6**), whose experimental data of the various intermediates as well as of the final compounds are hereinafter reported.

Compound 5

(*Z*)-Ethyl 4-(4-bromophenyl)-4-hydroxy-2-oxobut-3-enoate (27)

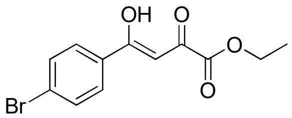

Mol. Wt.: 299.12

^{1}H NMR (500 MHz, $CDCl_3$): δ ppm 7.85 (d, 2H, *J* = 8.8 Hz, H-3′,5′), 7.63 (d, 2H, *J* = 8.8 Hz, H-2′,6′), 7.04 (s, 1H, -CH=), 4.38 (q, 2H, *J* = 7.4 Hz, -C*H*$_2$CH$_3$), 1.38 (t, 3H, *J* = 7.4 Hz, -CH$_2$C*H*$_3$). R_f = 0.82 (TLC: CH_2Cl_2/CH_3OH 95:5). Sticky reddish-brown solid.

Ethyl 5-(4-bromophenyl)-1*H*-pyrazole-3-carboxylate (29)

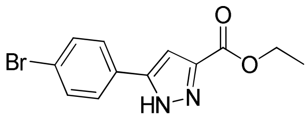

Mol. Wt.: 295.13

^{1}H NMR (500 MHz, $CDCl_3$): δ ppm 7.57 (d, 2H, *J* = 8.8 Hz, H-2′,6′), 7.51 (d, 2H, *J* = 8.8 Hz, H-3′,5′), 6.95 (s, 1H, Pyr*H*-4), 4.24 (q, 2H, *J* = 6.9 Hz, -C*H*$_2$CH$_3$), 1.25 (t, 3H, *J* = 6.9 Hz, -CH$_2$C*H*$_3$). R_f = 0.35 (TLC: Hexane/EtOAc 7:3). Yellowish solid; M.p. = 53–54 °C.

5-(4-Bromophenyl)-1*H*-pyrazole-3-carboxylic acid (31)

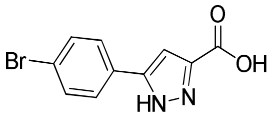

Mol. Wt.: 267.08

^{1}H NMR (500 MHz, Acetone-*d*$_6$): δ ppm 7.86 (d, 2H, *J* = 8.0 Hz, H-2′,6′), 7.62 (d, 2H, *J* = 8.0 Hz, H-3′,5′), 7.27 (bs, 1H, -COOH), 7.24 (s, 1H, Pyr*H*-4). R_f = 0.12 (TLC: 1% HCOOH in CH_2Cl_2/CH_3OH 95:5). Beige powder; M.p. = 147–149 °C.

(*E*)-Ethyl 3-(4-(5-(4-bromophenyl)-1*H*-pyrazole-3-carboxamido)phenyl)acrylate (33)

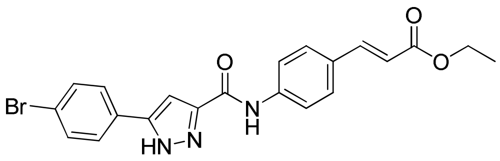

Mol. Wt.: 440.29

^{1}H NMR (500 MHz, DMSO-*d*$_6$): *δ* ppm 13.87 (bs, 1H, PyrN*H*), 10.31 (bs, 1H, -CON*H*), 7.90 (d, 2H, *J* = 8.4 Hz, Ar*H*), 7.80 (d, 2H, *J* = 8.3 Hz, Ar*H*), 7.77-7.65 (m, 4H, Ar*H*), 7.59 (d, 1H, *J* = 15.8 Hz, ArC*H*=), 7.26 (s, 1H, Pyr*H*-4), 6.53 (d, 1H, *J* = 15.8 Hz, =C*H*CO), 4.17 (q, 2H, *J* = 7.1 Hz, -C*H*$_2$CH$_3$), 1.24 (t, 3H, *J* = 7.1 Hz, -CH$_2$C*H*$_3$).
^{13}C NMR (125 MHz, DMSO-*d*$_6$): *δ* ppm 170.3 (-COOEt), 165.1 (-CONH), 147.8 (C3), 143.8 (ArCH=), 140.9 (C5), 132.4, 130.1, 129.4, 128.1, 125.1, 121.9 (C-Br), 120.7, 118.1, 106.2 (=CHCO), 104.0 (C4), 61.1 (CH$_2$), 13.2 (-CH$_2$CH$_3$). R$_f$ = 0.65 (TLC: CH$_2$Cl$_2$/CH$_3$OH 95:5). Yellow powder; M.p. = 177–180 °C.

(*E*)-3-(4-(5-(4-Bromophenyl)-1*H*-pyrazole-3-carboxamido)phenyl)acrylic acid (35)

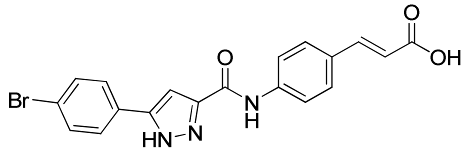

Mol. Wt.: 412.24
^{1}H NMR (500 MHz, DMSO-*d*$_6$): *δ* ppm 10.53 (bs, 1H, -CON*H*), 7.91 (d, 2H, *J* = 8.8 Hz, Ar*H*), 7.81 (d, 2H, *J* = 8.3 Hz, Ar*H*), 7.79 (d, 2H, *J* = 8.8 Hz, Ar*H*), 7.65 (d, 2H, *J* = 8.3 Hz, Ar*H*), 7.53 (d, 1H, *J* = 16.1 Hz, ArC*H*=), 7.22 (s, 1H, Pyr*H*-4), 6.44 (d, 1H, *J* = 16.1 Hz, =C*H*CO).
^{13}C NMR (125 MHz, DMSO-*d*$_6$): *δ* ppm 168.2 (-COOH), 164.5 (-CONH), 147.9 (C3), 144.0 (ArCH=), 141.1 (C5), 132.3, 129.9, 129.3, 127.8, 125.0, 121.8 (C-Br), 120.5, 118.0, 106.0 (=CHCO), 104.3 (C4). R$_f$ = 0.20 (TLC: 2% HCOOH in CH$_2$Cl$_2$/CH$_3$OH 95:5). Beige powder; M.p. = 198–201 °C.

(*E*)-5-(4-Bromophenyl)-*N*-(4-(3-(hydroxyamino)-3-oxoprop-1-enyl)phenyl)-1*H*-pyrazole-3-carboxamide (5)

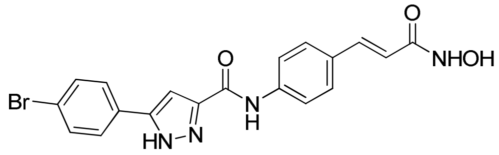

Mol. Wt.: 427.25
^{1}H NMR (500 MHz, DMSO-*d*$_6$): *δ* ppm 10.56 (bs, 1H, -CON*H*), 7.89 (d, 2H, *J* = 8.8 Hz, Ar*H*), 7.80 (d, 2H, *J* = 8.3 Hz, Ar*H*), 7.77 (d, 2H, *J* = 8.8 Hz, Ar*H*), 7.64 (d, 2H, *J* = 8.3 Hz, Ar*H*), 7.50 (d, 1H, *J* = 15.9 Hz, ArC*H*=), 7.19 (s, 1H, Pyr*H*-4), 6.43 (d, 1H, *J* = 15.9 Hz, =C*H*CO).
^{13}C NMR (125 MHz, DMSO-*d*$_6$): *δ* ppm 168.4 (-CONHOH), 164.1 (-CONH), 148.0 (C3), 143.6 (ArCH=), 141.0 (C5), 132.1, 129.8, 129.4, 128.0, 125.1, 121.7 (C-Br), 120.5, 118.1, 107.1 (=CHCO), 104.2 (C4). R$_f$ = 0.17 (TLC: 2% HCOOH in EtOAc/CH$_3$OH 8:2). Beige powder; M.p. = 222–224 °C. %). Anal. Calcd for $C_{19}H_{15}BrN_4O_3$: C, 53.41; H, 3.54; N, 13.11. Found: C, 53.47; H, 3.51; N, 13.15.

Compound 6

Ethyl 2-(3-hydroxy-1*H*-inden-2-yl)-2-oxoacetate (37)

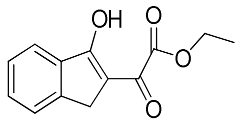

Mol. Wt.: 232.23

^{1}H NMR (500 MHz, $CDCl_3$): δ ppm 7.87 (d, 1H, *J* = 7.9 Hz, Ph*H*), 7.65 (t, 1H, *J* = 7.5 Hz, Ph*H*), 7.55 (d, 1H, *J* = 7.9 Hz, Ph*H*), 7.44 (t, 1H, *J* = 7.5 Hz, Ph*H*), 4.42 (q, 2H, *J* = 7.1 Hz, -CH_2CH_3), 4.00 (s, 2H, Ind-CH_2), 1.43 (t, 3H, *J* = 7.1 Hz, -CH_2CH_3). R_f = 0.74 (TLC: CH_2Cl_2/CH_3OH 95:5). Sticky brownish solid.

1,4-Dihydroindeno[1,2-*c*]pyrazole-3-carbohydrazide (38)

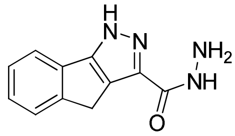

Mol. Wt.: 214.22

^{1}H NMR (500 MHz, DMSO-d_6): δ ppm 13.36 (bs, 1H, PyrN*H*), 9.91 (bs, 1H, CON*H*-), 9.35 (bs, 1H, CON*H*-), 7.61 (bs, 1H, Ph*H*), 7.53 (d, 1H, *J* = 7.3 Hz, Ph*H*), 7.34 (t, 1H, *J* = 7.3 Hz, Ph*H*), 7.26 (t, 1H, *J* = 7.3 Hz, Ph*H*), 4.59 (bs, 2H, -NH_2), 3.79 (bs, 2H, Ind-CH_2).

^{13}C NMR (125 MHz, DMSO-d_6): δ ppm 160.3 (-*C*ONHNH$_2$), 148.2, 134.4, 127.5, 126.9, 126.7, 126.6, 126.0, 119.7, 110.0, 29.3 (CH_2). R_f = 0.11 (TLC: 2% HCOOH in CH_2Cl_2/CH_3OH 95:5). Brown powder; Mp >250 °C.

1,4-Dihydroindeno[1,2-*c*]pyrazole-3-carboxylic acid (39)

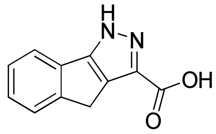

Mol. Wt.: 200.19

^{1}H NMR (500 MHz, CD_3OD): δ ppm 7.82 (d, 1H, *J* = 8.4 Hz, Ph*H*), 7.70 (d, 1H, *J* = 8.4 Hz, Ph*H*), 7.49 (dt, 1H, J_o = 8.4 Hz and J_m = 0.8 Hz, Ph*H*), 7.43 (dt, 1H, J_o = 8.4 Hz and J_m = 1.4 Hz, Ph*H*), 2.88 (AB-system, 2H, *J* = 15.4 Hz, Ind-CH_2).

^{13}C NMR (125 MHz, DMSO-d_6): δ ppm 161.6 (-COOH), 148.4, 134.2, 127.6, 127.4, 127.0, 126.7, 119.7, 29.4 (CH_2). R_f = 0.26 (TLC: 2% HCOOH in CH_2Cl_2/CH_3OH 95:5). Dark orange powder; M.p. >250 °C.

(*E*)-Ethyl 3-(4-(1,4-dihydroindeno[1,2-*c*]pyrazole-3-carboxamido)phenyl)acrylate (40)

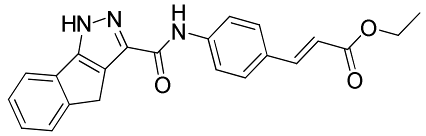

Mol. Wt.: 373.40

^{1}H NMR (500 MHz, DMSO-d_6): δ ppm 13.74 (bs, 1H, PyrN*H*), 10.32 (bs, 1H, -CON*H*), 7.93 (d, 1H, *J* = 7.9 Hz, Ar*H*), 7.76 (d, 1H, *J* = 16.1 Hz, ArC*H*=), 7.68 (d, 2H, *J* = 8.8 Hz, Ar*H*), 7.59 (2d, 3H, J_1 = 8.8 Hz and J_2 = 7.9 Hz, Ar*H*), 7.38 (t, 1H, *J* = 7.3 Hz, Ar*H*), 7.30 (dt, 1H, J_o = 7.9 Hz and J_m = 1.0 Hz, Ar*H*), 6.52 (d, 1H, *J* = 16.1 Hz, =C*H*CO), 4.16 (q, 2H, *J* = 7.4 Hz, -CH_2CH$_3$), 3.74 (s, 2H, Ind-CH_2), 1.24 (t, 3H, *J* = 7.4 Hz, -CH$_2$CH_3).

^{13}C NMR (125 MHz, DMSO-d_6): δ ppm 166.9 (-*C*OOEt), 161.1 (-*C*ONH), 151.0, 149.3, 144.5 (Ar*C*H=), 141.5, 141.0, 131.6, 130.6, 129.5, 127.4, 126.9, 120.3, 119.4, 116.7 (=*C*HCO), 60.3 (-*C*H$_2$CH$_3$), 29.7 (Ind-*C*H$_2$), 14.7 (*C*H$_3$). R_f = 0.58 (TLC: CH_2Cl_2/CH_3OH 95:5). Brownish powder; M.p. = 164–168 °C.

(*E*)-3-(4-(1,4-Dihydroindeno[1,2-*c*]pyrazole-3-carboxamido)phenyl)acrylic acid (41)

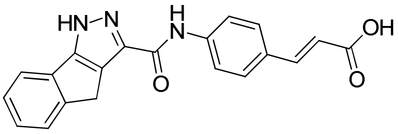

Mol. Wt.: 345.35

^{1}H NMR (500 MHz, DMSO-d_6): δ ppm 13.77 (bs, 1H, PyrN*H*), 13.61 (bs, 1H, -COO*H*), 10.12 (bs, 1H, -CON*H*), 7.99 – 7.78 (m, 1H, Ar*H*), 7.65 (d, 2H, *J* = 8.7 Hz, Ar*H*), 7.63 - 7.57 (m, 1H, Ar*H*), 7.49 (d, 1H, *J* = 16.1 Hz, ArC*H*=), 7.37 (t, 1H, *J* = 7.8 Hz, Ar*H*), 7.35 (d, 2H, *J* = 8.7 Hz, Ar*H*), 7.30 (t, 1H, *J* = 7.9 Hz, Ar*H*), 6.43 (d, 1H, *J* = 16.1 Hz, =C*H*CO), 3.71 (s, 2H, Ind-CH_2).

^{13}C NMR (125 MHz, DMSO-d_6): δ ppm 168.2 (-*C*OOH), 164.7 (-*C*ONH), 154.8, 149.2, 144.1 (Ar*C*H=), 141.6, 138.3, 130.8, 129.8, 129.4, 127.4, 127.0, 120.4, 119.3, 117.7 (=*C*HCO), 29.4 (Ind-*C*H$_2$). R_f = 0.58 (TLC: 2% HCOOH in CH_2Cl_2/CH_3OH 95:5). Rusty powder; M.p. >250 °C.

(*E*)-N-(4-(3-(hydroxyamino)-3-oxoprop-1-enyl)phenyl)-1,4-dihydroindeno[1,2-*c*]pyrazole-3-carboxamide (6)

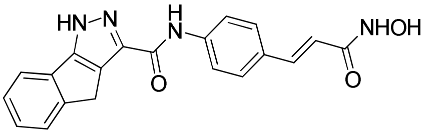

Mol. Wt.: 360.37

^{1}H NMR (500 MHz, DMSO-d_6): δ ppm 13.88 (bs, 1H, PyrN*H*), 9.98 (bs, 1H, -CON*H*), 8.00 – 7.80 (m, 1H, Ar*H*), 7.67 (d, 2H, *J* = 8.8 Hz, Ar*H*), 7.65 - 7.53 (m, 1H, Ar*H*), 7.50 (d, 1H, *J* = 16.1 Hz, ArC*H*=), 7.36 (t, 1H, *J* = 7.9 Hz, Ar*H*), 7.32 (d, 2H, *J* = 8.8 Hz, Ar*H*), 7.29 (t, 1H, *J* = 7.9 Hz, Ar*H*), 6.40 (d, 1H, *J* = 16.1 Hz, =C*H*CO), 3.69 (s, 2H, Ind-CH_2).

^{13}C NMR (125 MHz, DMSO-d_6): δ ppm 169.5 (-*C*ONHOH), 165.1 (-*C*ONH), 155.0, 149.1, 143.9 (Ar*C*H=), 141.5, 138.5, 130.8, 129.9, 129.4, 127.2, 126.9, 120.6, 119.4, 117.8 (=*C*HCO), 29.6 (Ind-*C*H$_2$). R_f = 0.58 (TLC: 2% HCOOH in EtOAc/CH_3OH 8:2). Rusty powder; M.p. >250 °C. Anal. Calcd for $C_{20}H_{16}N_4O_3$: C, 66.66; H, 4.48; N, 15.55. Found: C, 66.70; H, 4.46; N, 15.58.
